# Extracellular vesicle-based liquid biopsy biomarkers and their application in precision immuno-oncology

**DOI:** 10.1186/s40364-023-00540-2

**Published:** 2023-11-17

**Authors:** Karama Asleh, Valerie Dery, Catherine Taylor, Michelle Davey, Marie-Ange Djeungoue-Petga, Rodney J. Ouellette

**Affiliations:** 1 Atlantic Cancer Research Institute, Moncton, New Brunswick Canada; 2 Department of Chemistry and Biochemistry, Université de Moncton, Moncton, New Brunswick Canada; 3 Dr Georges L. Dumont University Hospital, Vitalite Health Network, Moncton, New Brunswick Canada; 4 Beatrice Hunter Cancer Research Institute, Halifax, Nova Scotia Canada

**Keywords:** Extracellular vesicles, Exosomes, Liquid biopsy, Biomarkers, Tumor microenvironment, Intercellular crosstalk, Multi-omics, Machine learning, Immunotherapy, Precision immuno-oncology

## Abstract

While the field of precision oncology is rapidly expanding and more targeted options are revolutionizing cancer treatment paradigms, therapeutic resistance particularly to immunotherapy remains a pressing challenge. This can be largely attributed to the dynamic tumor-stroma interactions that continuously alter the microenvironment. While to date most advancements have been made through examining the clinical utility of tissue-based biomarkers, their invasive nature and lack of a holistic representation of the evolving disease in a real-time manner could result in suboptimal treatment decisions. Thus, using minimally-invasive approaches to identify biomarkers that predict and monitor treatment response as well as alert to the emergence of recurrences is of a critical need. Currently, research efforts are shifting towards developing liquid biopsy-based biomarkers obtained from patients over the course of disease. Liquid biopsy represents a unique opportunity to monitor intercellular communication within the tumor microenvironment which could occur through the exchange of extracellular vesicles (EVs). EVs are lipid bilayer membrane nanoscale vesicles which transfer a plethora of biomolecules that mediate intercellular crosstalk, shape the tumor microenvironment, and modify drug response. The capture of EVs using innovative approaches, such as microfluidics, magnetic beads, and aptamers, allow their analysis via high throughput multi-omics techniques and facilitate their use for biomarker discovery. Artificial intelligence, using machine and deep learning algorithms, is advancing multi-omics analyses to uncover candidate biomarkers and predictive signatures that are key for translation into clinical trials. With the increasing recognition of the role of EVs in mediating immune evasion and as a valuable biomarker source, these real-time snapshots of cellular communication are promising to become an important tool in the field of precision oncology and spur the recognition of strategies to block resistance to immunotherapy. In this review, we discuss the emerging role of EVs in biomarker research describing current advances in their isolation and analysis techniques as well as their function as mediators in the tumor microenvironment. We also highlight recent lung cancer and melanoma studies that point towards their application as predictive biomarkers for immunotherapy and their potential clinical use in precision immuno-oncology.

## Background

Despite advances in molecular diagnostics and newer therapeutic options made available over the past decade, survival benefits are limited to a subset of cancer patients [1]. Resistance even to the most effective immunotherapies is still observed over an extended period and can further develop as the tumor evolves, posing a major challenge to the process of biomarker development [2]. Mounting evidence attributes this challenge to the dynamic nature of tumor progression along with the diverse milieu of the tumor microenvironment (TME) involving epithelial, stromal, and immune cells components [3]. Thus, there is a pressing clinical need to identify biomarkers able to alert to the emergence of recurrences and monitor treatment response among patients in a real-time fashion.

Liquid biopsy-based biomarkers have garnered special interest in the field of precision oncology as they possess several advantages over tissue-based biomarkers including their minimally-invasive nature, rapid turnaround collection time, convenience and feasibility to obtain several samples from patients across key time points for early diagnosis, prognosis and monitoring treatment response [4, 5]. Furthermore, liquid biopsies can be performed on a relatively small amount of blood, urine, saliva, cerebral spinal fluid, and pleural effusion to identify circulating biomarkers [6]. The liquid biopsy specimen contains a sampling of heterogenous constituents that are potentially representative of the underlying specific patterning of the disease state [7]. Accordingly, the field of biomarker development is rapidly shifting towards exploring the utility of these real-time snapshots of tumors from bodily fluids to allow for a holistic representation of the evolving disease course essential to inform better treatment decisions [5] in lieu of initial tissue biopsy.

Recent approaches have been utilized to identify clinically useful biomarkers focusing mainly on circulating tumor cells (CTCs) and circulating tumor DNA (ctDNA) [8–10]. CTCs have the potential to provide critical information that aids the development of diagnostic, prognostic, and predictive biomarkers for treatment response in a real-time manner [5, 8, 11]. For instance, several genetic and epigenetic profiles of CTCs isolated from cancer patients have been linked to prognosis [5, 8]. When compared to CTCs, ctDNA is gaining a higher impetus in the clinical setting as its prognostic significance and ability to serially monitor residual disease during treatment have been demonstrated across several cancer types [5, 9, 12, 13]. Clinical trials with immunotherapy have further demonstrated the predictive capacity of ctDNA as a biomarker that can be linked to survival benefit [14, 15]. In addition, ctDNA has an ability to detect actionable mutations overlooked by tissue-based genotyping to allow matching advanced cancers to targeted therapies [16–20].

Nonetheless, CTCs and ctDNA are associated with multiple challenges that limit their clinical application. CTCs are characterized by a short life span, low numbers and concentration, dynamic heterogeneity, often rely on epithelial markers for isolation that exclude CTCs with mesenchymal phenotypes, and require advanced technologies such as microfluidic devices and post-enrichment strategies to improve sensitivity [5, 8, 21, 22]. Analysis of ctDNA often requires a larger volume of blood sample as it only accounts for 0.1–10% of the total circulating cell-free DNA (cfDNA) [5, 23] and mutations identified can also be reflective of non-malignant cells leading to false positive results [8]. Furthermore, standardization of ctDNA methodologies and establishing their analytical validity are still required prior to their incorporation in the clinic [10].

The complexity of the TME and its underlying role in inducing tumor therapeutic resistance highlights the need to integrate this level of information in the process of biomarker development. Indeed, liquid biopsy represents a unique opportunity to real monitor intercellular communication between the tumor and its surroundings which could occur through the exchange of extracellular vesicles (EVs). These are nanoscale vesicles with a lipid bilayer membrane that are secreted by all cells including cancer cells, and act as key mediators of intercellular communication [5, 24]. EVs contain a diverse cargo of biomolecules such as DNA, RNA including mRNA, microRNA (miRNA), long non-coding RNA (lncRNA), proteins, metabolites and lipids which represent the heterogeneity of their parental cells, making them a great source for biomarker discovery [25]. EVs have several additional advantages over ctDNA and CTCs as they are present in profuse quantities in biofluids which make them easier to obtain, are comparatively stable due to their lipid bilayers and can be stored at − 80 °C for a relatively longer period with a preservation to their morphology and content [26, 27]. As such, research efforts in the field of liquid-biopsy based biomarkers are being increasingly focused on EVs as important mediators that govern interactions between various cell types within the TME. Furthermore, a growing number of studies are describing their role in a myriad of critical biological processes for cancer development that span establishing a premetastatic niche, potentiating tumor progression, inducing tumor angiogenesis, regulating immune response, and remodeling metabolic activity [5, 28–32].

In this review, we discuss the emerging role of EVs for biomarker development describing current advances in their isolation techniques and analysis, their function as mediators of intercellular crosstalk in the TME, and their clinical application as predictive biomarkers for immunotherapy providing insights from recent lung cancer and melanoma studies and highlighting their potential use in clinical trials in the era of precision immuno-oncology.

## Overview of EV subsets as a source for biomarker discovery

**Fig. 1:**
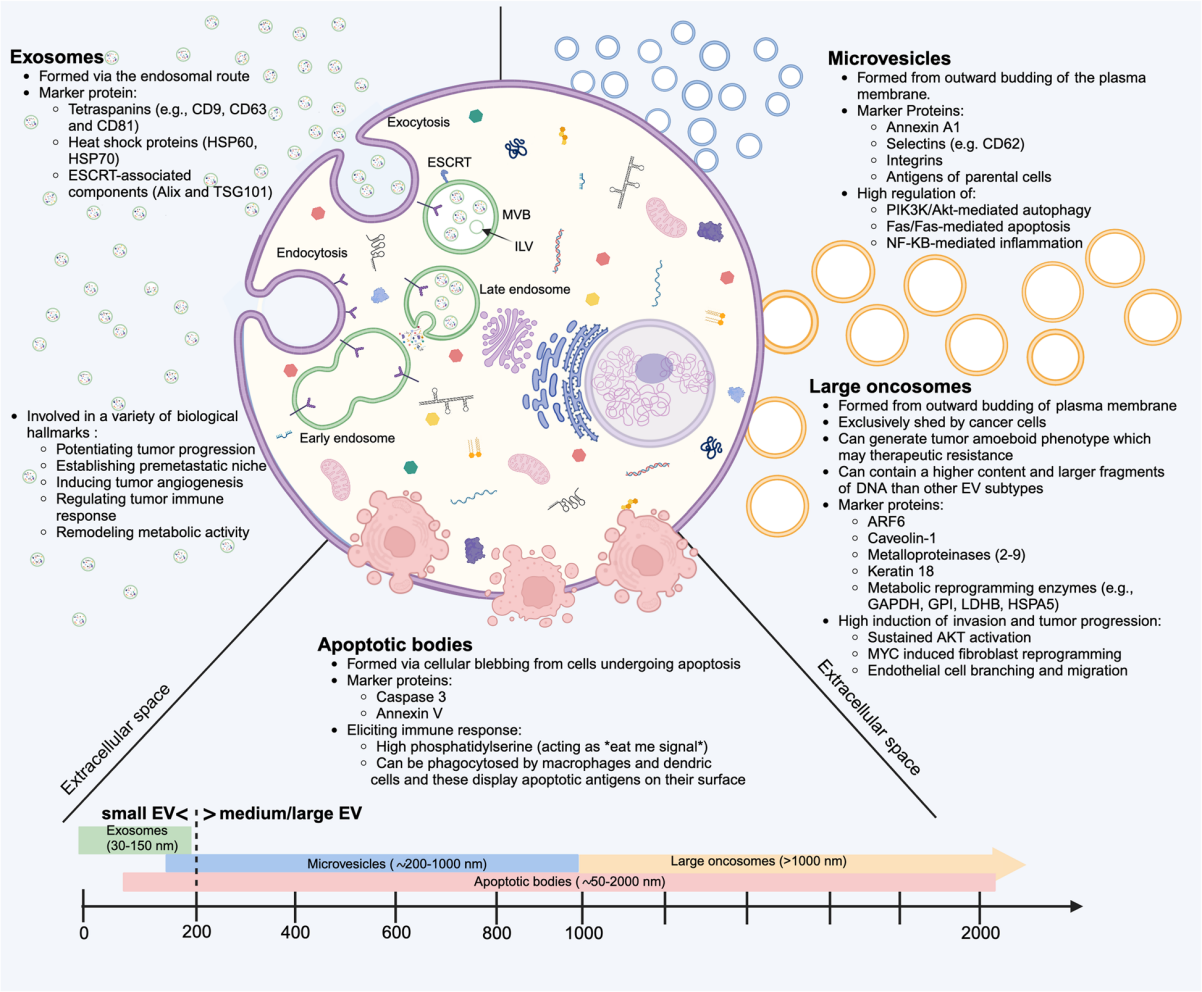
Overview of key characteristics related to biogenesis, biomarker expression, and cellular function of the major extracellular vesicle subsets of exosomes, microvesicles, apoptotic bodies and large oncosomes. Abbreviations: EV, extracellular vesicles; ESCRT endosomal sorting complex required for transport; ILV, intraluminal vesicle; MVB, multivesicular body. Figure was created with BioRender.com

EVs were originally viewed as molecular “garbage bins” that assist in removing undegraded endosomal or lysosomal components from cells. However, the recognition that their cargoes can be taken up by diverse recipient cells have established their crucial role in mediating various forms of intercellular communication [5]. EVs consist of a heterogeneous group which according to the International Society of Extracellular Vesicles (ISEV) can be classified into different subpopulations based on their size, morphology, and route of biosynthesis [33]. These mainly include exosomes which are small-sized EVs (∼30–150 nm in diameter), ectosomes consisting of microvesicles (∼200–1000 nm) and large oncosomes (> 1000 nm), apoptotic bodies (∼50–2000 nm) [34], as well as less abundant populations including migrasomes [35]. Other subsets such as mitochondrial-derived vesicles have been recently described [36]. Key characteristics of the major extracellular vesicle subsets are displayed in Fig. 1.

Exosome are generated through the endosomal route which starts with invagination of plasma membrane, formation of early-sorting endosomes that contain cell surface and soluble extracellular proteins and maturation into late-sorting endosomes which invaginate to give rise to intraluminal vesicles that contain cytoplasmic proteins and other constituents. Subsequently, multivesicular bodies (MVBs) containing intraluminal vesicles are formed, transported to the plasma membrane, and released as exosomes through exocytosis [37]. The process of cargo sorting during exosome formation is unique and occurs in both endosomal sorting complex required for transport (ESCRT)-dependent and -independent manners [38–40]. Some studies have reported that exosomes not only reflect the contents of their parental cells but could also differ across tumors and various pathophysiological states making them a hugely invaluable resource for biomarker discovery. For instance, some RNA components in exosomes have been shown to be distinct from their parental cells suggesting that different pathways can be involved in the various steps of cargo sorting which result in the coexistence of distinct subpopulations of MVBs and intraluminal vesicles [41]. In addition, the composition of cell surface proteins within exosomes could play a key role in mediating their effect and defining exosomal expression patterns that can be characteristic of specific cancer types and disease stages [41]. Key proteins reported to be found in exosomes are tetraspanins (e.g., CD9, CD63 and CD81), heat shock proteins (HSP60, HSP70) and ESCRT-associated components (Alix and TSG101) that could be used as fingerprints for certain tumors [42]. For instance, CD63 is highly expressed in ovarian cancer when compared to lung cancer [41], which is in turn characterized by a high expression of exosomal CD91 [43] and several other EV markers [44]. As such, exosomes display a promising tool for developing biomarkers that reflect the dynamic and heterogenous nature of cancer [45].

Unlike exosomes, ectosomes are comprised of microvesicles (MVs), large oncosomes and apoptotic bodies that are generated by outward budding of the plasma membrane, which is pinched off and released to the extracellular space [32]. The mechanism by which MVs are formed and shed at the cell surface is not fully understood but has been suggested to involve a membrane asymmetry and cytoskeleton rearrangements [45]. Several proteins can mediate these processes by regulating the lipid composition and distribution in the plasma membrane, adding contractile forces that lead to membrane bending, promoting actin-myosin contraction through activation of RhoA/ROCK signaling, translocation of amino-phospholipids in an adenosine triphosphate (ATP)-dependent manner and activity of enzymes such as flippases stimulated by high concentration of calcium ions [45, 46]. In general, MVs are comprised of lipids and proteins derived from parental cell membrane components, secretory proteins, and genetic material transmitted in the cytoplasm of the cell of origin [47]. For instance, MVs derived from apoptotic cells are characterized by CD31/annexin V, while MVs generated by endothelial cells engaging in angiogenesis carry CD105 or CD62E and those produced during neutrophil chemotaxis and inflammation may carry CD66b [47]. Active substances are transferred by MVs to target cells through receptor ligand binding, direct fusion, and endocytosis where they carry out diverse functions such as regulation of gene expression, protein synthesis and other various biological effects that can change depending on pathological conditions [47]. MV release has been shown to increase under hypoxic conditions which is known to be an important external stimulus for the dissemination of malignant behaviors between tumor cells [48]. Furthermore, three main pathways have been described to be highly regulated by MVs and their active molecules including (a) phosphoinositide 3-kinase (PI3K)/ Ak strain transforming (Akt)-mediated autophagy, (b) Fas/Fas ligand-mediated apoptosis and (c) NF-κB–mediated inflammation [47]. In this context, MVs could aid in identifying biomarkers linked to cancer prognosis and therapeutic response.

Apoptotic bodies are formed via cellular blebbing after programmed cell death and the disassembly of an apoptotic cell into subcellular fragments which may contain components such as micronuclei, chromatin remnants, cytosol portions, degraded proteins, and DNA fragments [49]. While they have been referred to as “little sealed sacs” that are a hallmark of apoptosis functioning to remove cell debris, apoptotic bodies have been described to also regulate intercellular communication [48]. Dying cells can release apoptotic bodies that can be more abundant than exosomes and MVs under certain conditions and may contain diverse biomolecules including DNA, miRNA, and ribosomal RNA and thus deliver useful materials to recipient cells [49]. Due to their apoptotic origin, apoptotic bodies retain phosphatidylserine (PS) as a common surface marker which may act as an “eat me” signal to facilitate the recognition and uptake of apoptotic cells by phagocytes after their release [49]. Apoptotic bodies can be phagocytosed by macrophages and dendritic cells (DCs) which can display apoptotic antigens on their surface to facilitate immune response [48]. As such, they may play a potential role in activating the immune system, a property that has been exploited for further investigation in vaccine development and immunotherapy [49, 50].

Large oncosomes have been identified relatively recently compared to other EV subpopulations, but they are unique in being exclusively shed by cancer cells [51]. Their biogenesis mechanisms are akin to MVs as both originate from the plasma membrane, although some data suggest that a higher amount of cholesterol in the plasma membrane results in an increased shedding of large oncosomes [51]. In addition, large oncosomes have been linked to the generation of tumor amoeboid phenotype which may confer therapeutic resistance thorough several mechanisms such as activation of RhoA/ROCK, silencing of a cytoskeletal regulator called DIAPH3, increased intracellular calcium influx, and cytoskeleton reorganization induced by extracellular matrix (ECM) degradation products [51, 52]. Among ectosomes, large oncosomes are unique in the field of biomarker discovery due to their properties of being exclusive to cancer cells and having a large size which can contain many tumor-derived molecules that mediate several signaling processes [53]. For instance, they can contain up to 7-fold more DNA with large DNA fragments ranging from 100 Kbps to 2 Mbps as observed in an analysis of plasma obtained from prostate cancer patients [54]. Whole genome sequencing analysis in this study revealed that the DNA of large oncosomes covered the entire reference genome of their donor cells and was reflective of their mutational status and copy number alterations [54]. As such, large oncosomes cargo displays a promising resource for developing more comprehensive prognostic and predictive cancer biomarkers representative of multiple pathways in the TME while covering the whole cancer genome [55]. Key molecules that have been specifically described as more characteristics of large oncosomes include ARF6 involved in the contraction of actomyosin, caveolin-1, metalloproteinases (MMPs) 2–9, keratin 18, and several enzymes involved in metabolic reprogramming such as GAPDH, GPI, LDHB, HSPA5 [51]. Of note, pathways that confer tumor progression, invasion, and therapeutic resistance such as sustained AKT activation, MYC induced fibroblast reprogramming, endothelial cell branching and migration have been described as being specific to large oncosomes, suggesting that investigating this specific form of EV might be more relevant to the identification of biomarkers related to therapeutic resistance [56].

## Current methodologies for the isolation of EVs from liquid biopsies

To date, several techniques of EV isolation suitable for biomarker development have been proposed. However, the application of these techniques in the clinic is still limited due to several factors, in particular the lack of optimized and standardized protocols that maintain high purity and accurate quantification of EVs [57, 58]. The heterogeneity in physical and biochemical properties of EVs and their presence within a mix of various cellular debris and components in body fluids influence their effective isolation [33, 59]. Thus, many conventional techniques often exploit only one or few EV characteristics such as size, morphology, density, or surface biomarkers contributing to analytical variability issues [60]. Classical methods for EV isolation include ultracentrifugation, ultrafiltration, size exclusion chromatography (SEC), and polymer precipitation which are often associated with limitations, such as low purity, contamination, and EV loss and breakage that affect their downstream analysis and accurate quantification [5]. To improve the performance of EV isolation techniques, several modifications to existing conventional methods including combination approaches have been implemented [5] and newer technologies such as microfluidic chips and magnetic beads that hold the potential to progress the field of liquid biopsy biomarkers have been recently developed [22, 61–63].

Ultracentrifugation is the most widely used technique for EV isolation which is based on sedimentation velocity that exploits differential size, shape, and density. Modifications to ultracentrifugation protocols that include high rotor speed and extended centrifugation time have been shown to improve EV purity for exosomal content and vesicle yield respectively, which can be particularly important when handling small volumes of clinical samples [64]. However, it should be noted that a very lengthy centrifugation time can result in excessive contamination of soluble proteins to vesicular material and low specificity **[**64**]**. Thus, the selection of isolation protocol should be tailored to the scientific question tested as recommended by ISEV [33].

Ultrafiltration techniques which apply membrane filters with specified molecular weight cut-offs are also used. However, since these techniques often use pressure, breakage or deformation of the vesicles can occur which affect their downstream analysis [5]. Furthermore, large structures can disintegrate and pass through the filter along with small vesicles resulting in a lower specificity for EV isolation [33]. Thus, filter types can significantly affect recovery [65] and ultrafiltration techniques are commonly recommended to be paired with another method such as SEC to improve EV isolation specificity with minimal non-EV components [66]. In SEC methods, EVs are eluted in earlier fractions than other components in the sample based on their exclusion from pores within the stationary phase. These methods have been utilized in isolating tumor-derived EVs (TEVs) that have effect on T cell suppression from ascites of ovarian cancer patients as confirmed by Western immunoblotting [67]. Furthermore, the combination of SEC and ultracentrifugation has been shown to improve EV yield and enrich for EV biomarkers associated with nephrotic syndrome from urine of patients with nephrotic-range proteinuria when compared to nanomembrane ultrafiltration or ultracentrifugation only [68]. SEC methods are characterized as ‘intermediate recovery, intermediate specificity’ according to ISEV, but may co-isolate certain lipoproteins along with EVs and thus sequential techniques may be needed to overcome this limitation [33]. Variations such as sequential filtration that utilizes a 3-step protocol of dead-end pre-filtration, tangential flow filtration, and low-pressure track-etched membrane filtration, have been shown to result in better purity and integrity with protein isolates verified by mass spectrometry (MS), nanoparticle tracking analysis (NTA) and electron microscopy [69, 70]. Additional methods have been developed to achieve better recovery or specificity including asymmetric flow-field flow fractionation [71], variations on SEC [72, 73], ion exchange chromatography [74, 75] and others [33].

Precipitation methods that utilize polymers such as polyethylene glycol (PEG) are one of the most popular methods to isolate EVs [33]. These techniques are often classified as ‘high recovery, but low specificity’ according to ISEV due to concerns regarding some precipitation studies where the results were due to the presence of residual contaminants rather than the function of the precipitated EVs [76]. However, some isolation kits such as ExoQuick PLUS and ExoQuick-TC PLUS (System Biosciences) can precipitate EVs from a wide range of biofluids with reduced carryover contaminants in a relatively short time and are compatible for use in downstream applications including RNA-seq, quantitative PCR (qPCR), MS, and Western blotting [77, 78]. EV purity and yield have been shown to be further enhanced when combining ExoQuick-TC PLUS with ultracentrifugation for isolating EVs from the serum of breast cancer patients [77]. Combined isolation techniques of PEG precipitation with SEC have further resulted in a better EV yield and protein recovery with a less time-consuming process [79]. Additional precipitation methods, such as lectin-based agglutination that precipitates EVs based on high binding affinity to oligosaccharide residues on EV membrane, have been further reported and used to describe miRNA urine profiles for the diagnosis of prostate cancer [80].

Non-specific binding, matrix contaminants and EV heterogeneity pose major limitations to the clinical application of classical EV isolation methods when processing routine clinical samples for the purpose of biomarker discovery. Thus, techniques that are characterized by higher efficiency and specificity for EV subtype capture would have the potential to be more easily translated into clinically actionable tests. Affinity-based assays including enzyme-linked immunosorbent assay (ELISA) are often categorized with a high specificity by ISEV as they are comparatively more selective in isolating and analyzing EVs based on the expression of membrane-bound surface markers that are either overly expressed on EVs in general or specific to an EV subtype [33]. While the specificity of affinity-based techniques can reduce the total recovery of EVs, specific subtypes of EVs may be enriched and isolated with high recovery relative to classical methods using techniques such as magneto-immunocapture which apply antibody-coated magnetic particles to selectively enrich for EV populations [33]. For instance, specific exosomal proteins and RNAs were recovered with a better yield from EVs isolated using immunoaffinity-based assays coupled with a selective immobilization of exosomes captured on a solid phase of magnetic beads when compared to conventional methods or other benchmark technologies [81]. Several other magneto-immunocapture techniques are characterized with utilizing different surface biomarkers to enrich for EVs such as CD63 [82], or other exosomal subset markers that could be relevant to specific tumor types such as A33+ colon cancer-derived EVs [83], and CD34+ blast-derived EVs in acute myeloid leukemia [84]. A recent study has integrated magnetic isolation with enhanced fluorescence measurement to develop a homogenous magneto-fluorescent exosome nanosensor that detects tumor derived exosomes by separating GPC-1 positive exosomes from plasma of breast cancer patients [85].

Immunoaffinity-based microfluidics are gaining momentum as they have a tremendous potential for clinical application due to several advantages including time-saving procedures, small consumption of samples and reagents, and increased efficiency in specifically trapping and separating small EVs in cancer patients’ liquid samples [61, 86]. Some of these microfluidic chips use magnetic nanoparticles coupled with antibodies to either common exosomal markers such as CD63 or to tumor-derived markers such as epithelial cellular adhesion molecule (EpCAM) and CA-125 [87, 88] which have demonstrated a diagnostic capacity in breast and ovarian cancers [87,88]. ExoChip, a commercially available CD63 antibody-coated microfluidic chip, has been shown to enable molecular profiling of exosomal microRNAs in serum from pancreatic cancer patients and was proposed as a potential clinical platform for biomarker discovery [89]. In addition, microfluidic immunochips have allowed a selective isolation of exosome subpopulations targeting a panel of markers (EpCAM, α-IGF-1R, CA125, CD9, CD81 and CD63) in non-small-cell lung cancer (NSCLC) from a minimal amount of plasma (30 μL) within ~ 100 minutes [41].

More recently, immunoaffinity-based microfluidics combined with filtration approaches such as ExoDIF [90] and Exo-ID chip [91] have been developed to allow both efficient capture and release of exosomes for downstream analysis. For instance, ExoDIF which introduces exosome-specific dual-patterned immunofiltration demonstrated better efficiency in capturing and releasing exosomes with higher purity from exosome-enriched blood samples of breast cancer when compared to the conventional ExoQuick kit, allowing a better downstream analysis to identify biomarkers that differentiate breast cancer patients from healthy donors **[**90**]**. Recent advancements in microfluidics have been further made in specifically identifying cancer-related exosome subpoulations in several cancer types. Sub-ExoProfile chip which comprises three cylindrical self-assembled nanopillars to capture CD81, EpCAM, and Her2-positive exosomes showed a high capture efficiency, even for exosomes with low expression of these surface markers, and was able to distinguish Her2+ from triple negative breast cancer subtypes in clinical serum samples [92]. Other microfluidic chips targeting specific exosome subpopulations have shown potential to be developed as early detection and diagnostic tools applicable to clinical samples, such as EVs on demand chip (EVOD) which incorporates anti-EpCAM/anti-epidermal growth factor receptor (EGFR) for NSCLC [93] and EV Click Chips purification system which integrates a multi-marker cocktail (anti-EpCAM, anti-ASGPR1, and anti-CD147) for hepatocellular carcinoma (HCC) [94].

While immune-based isolation techniques open new perspectives towards clinical applications, they are not devoid of challenges associated with high costs and being marker-dependent. As such, additional approaches that are not antibody-based which apply a simple, fast, efficient, and affordable technique are emerging. Recently, Chen et al described an ultrafast-isolation system, called EXODUS, which employs a nanoporous membrane to rapidly and continuously isolate exosomes with a high purity from biofluids by applying periodic negative pressure oscillations to allow small particles and fluids to pass through the filter and acoustofluidic streaming for resuspension of particles into the liquids [95]. This technique demonstrated a practical relevance for high-throughput exosomal RNA profiling using urine samples from 113 patients [95]. Other methods have utilized lipid nanoprobes, based on lipid layer labelling and magnetic enrichment, allowing rapid isolation of nanoscale EVs from plasma of NSCLC patients with subsequent identification of *EGFR* and Kirsten rat sarcoma virus *(KRAS)* mutations in downstream analysis [96]. A peptide affinity isolation method which uses Vn96, a synthetic peptide that can bind HSPs on the EV surface [97, 98], has demonstrated a high efficiency in isolating EVs from a minimum volume of plasma allowing analyses of proteins, small RNAs and DNA on the same clinical sample for multi-omics biomarker development [99]. Furthermore, the Vn96-based isolation method has shown a clinical utility in detecting *EGFR* mutations in plasma EVs from NSCLC patients with 100% specificity [100], identifying diagnostic small RNA biomarkers for pancreatic cancer [101], and developing an RNA-based diagnostic panel for prostate cancer using urinary EVs [102]. Additional isolation methods have been recently described such as acoustic-based microfluidics [25] which apply ultrasonic waves with forces that separate particles depending on their physical properties. Other variations that can sort EV subpopulations by combined electrical and acoustic forces have resulted in an efficient exosome recovery and high purity [103]. Innovative approaches that integrate a mobile ionic exchange platform with magnetic beads called ExoCAS-2 (exosome clustering and scattering) that enables a high exosome recovery and purity with a potential for a clinical application have been described [104]. The development of aptamer-based small-molecule sensors is an emerging approach which relies on the application of short oligonucleotides that can bind with a high affinity to specific molecules offering several advantages for biosensing in comparison to antibodies [105]. Aptamers have been recently integrated in thermophoretic enrichment assays which apply a laser heating to create a temperature gradient allowing for a rapid isolation and enrichment of tumor-derived exosomes [106]. This approach has shown a clinical potential in developing a plasma EV-based signature for prognosis and monitoring treatment response in metastatic breast cancer patients [107]. Methodologies for the isolation of EVs from liquid biopsies appear in Table 1.

**Table 1:** Methodologies for the isolation of extracellular vesicles from liquid biopsies

Technique	Principle	Advantages	Limitations
Ultracentrifugation	Sedimentation velocity	High purity; Widely used.	Lengthy process; Low yield; Require specialized equipment; Contamination of soluble proteins to vesicular material resulting in low specificity.
Ultrafiltration	Size-based (membrane filters with specified molecular weight cut-offs)	High purity; Easily applied.	Contamination of same-sized vesicles resulting in low specificity; Breakage or deformation of the vesicles can occur due to pressure.
Size exclusion chromatography	Size-based exclusion (EVs are eluted in earlier fractions based on their exclusion from pores within the stationary phase)	Relatively high yield; Easily applied.	Contamination of same-sized vesicles resulting in low specificity; Co-isolation of certain lipoproteins along with EVs which may require sequential techniques for separation.
Precipitation methods			
Polyethylene glycol	Precipitation	High yield and recovery; Popular; Easily applied.	Low specificity due to the presence of residual contaminants.
Commercialized reagent kits (Examples: ◦ ExoQuick PLUS ◦ ExoQuick-TC PLUS)	Precipitation	High yield and recovery; Easily applied; Ability to precipitate EVs from a wide range of biofluids with reduced carryover contaminants; Relatively short processing time; Compatibility for use in downstream applications.	Relatively low specificity, but recent developments have reduced carryover contaminants; High costs.
Immunoaffinity-based assays			
Magneto-immunocapture	Antibody-coated magnetic particles	High specificity; Selective enrichment for EV populations (mainly small EVs).	Marker dependent; High costs.
Microfluidic immunochips (Examples: ◦ ExoChip: CD63 antibody-coated microfluidic chip. ◦ ExoDIF and Exo-ID chip: immunoaffinity-based microfluidics combined with filtration approaches. ◦ Sub-ExoProfile chip: a microfluidic nanodevice with three self-assembled 3D nanopillars immobilized with capture antibodies for CD81, EpCAM, and Her2). ◦ EVs on demand chip (EVOD) and EV Click Chip: Click chemistry purification system integrating multimarker antibody cocktails for small EVs.	Antibody-coated microchannel chip (selective capture of small EV molecules)	High specificity; Time-saving procedures; Small consumption of samples and reagents; Increased efficiency in specifically trapping and separating small EVs; High compatibility for high-throughput analysis.	Marker dependent; High costs.
Others			
Size-based microfluidic chips (Examples: ◦ ExoTIC ◦ EXODUS	Physical properties (Nanoporous membrane for isolating small EVs of certain size range)	Time-saving procedures; Small consumption of samples and reagents; Increased efficiency in separating small EVs; High compatibility for high-throughput analysis.	Relatively low yield; Blockage of membrane pores can limit the continuous separation of small EVs. Recent developments with faster systems (such as those integrating periodic negative pressure oscillations and acoustofluidic streaming in EXODUS) are reducing these limitations.
Acoustic-based microfluidic chips	Physical properties (ultrasonic waves with forces to separate particles)	Rapid; Continuous and efficient separation of particles and EVs; Non-contact; Application to a wide range of particles and vesicles allowing EV sorting.	Design and manipulation of bioparticles and submicrometer particles for deterministic sorting.
Lipid nanoprobes	Lipid layer labelling and magnetic enrichment	Rapid; Efficient isolation of nanoscale EVs.	Low specificity; Contamination of other phospholipid membrane vesicles can occur.
Peptide affinity assays (Examples: ◦ Vn96: can bind heat shock proteins on EV surface)	Synthetic peptide binds molecules on the EV surface	Rapid; Inexpensive; High efficiency in isolating EVs from a minimum volume of liquid biopsy; Feasibility of simultaneous analyses of proteins, small RNAs and DNA.	Affinity for both EVs and circulating cell free DNA. The synthetic peptide must be removed from the sample prior to proteomic analysis.
Aptamer-based assays	Short oligonucleotides that can bind with high affinity to specific molecules targeting specific markers	Rapid; Binding targets with a higher affinity and better biosensing in comparison to antibodies; Capture of specific cancer biomarkers allowing enrichment of tumor-derived exosomes; Less expensive than antibodies.	Risk of degradation by nuclease activity; Limited reproducibility for small EV proteins in clinical setting; Need to develop standardized selection and modification strategies for highly performing aptamers.

*Abbreviations:* *EV *Extracellular vesicle, *CD* Cluster of differentiation, *EpCAM *Epithelial cellular adhesion molecule, *Her2 *Human epidermal growth factor receptor 2

Overall, several analytical factors such as the varying laboratory protocols used to perform EV isolation, their instrumentation, optimization, quality control procedures, and reproducibility assessment, still hinder the adoption of a standardized EV isolation method that can be used for clinically amenable biomarker discovery and validation.

## Key recent developments in analysis of EVs from liquid biopsy

Per the minimal information for studies of EV (MISEV) 2018 guidelines, it is critical to assess the results of isolation methods by quantitative measures of the source of EVs (e.g. volume of biofluid), their abundance, the presence of components associated with EVs generally or with EV subtypes, and the existence of non-vesicular co-isolated components in order to establish the validity of the isolation assay for biomarker development [33]. NTA is a widely used method for assessing the concentration and size distribution of particles in which the light scattered by EVs illuminated by a laser beam is recorded by a microscope camera and used to track their Brownian motion [108]. Other techniques for EV quantification and characterization include standard flow cytometry for larger EVs, high resolution flow cytometry for smaller EVs, resistive pulse sensing and tunable resistive pulse sensing for a wide range of sizes, electron microscopy (TEM and cryo-EM), atomic force microscopy, dynamic light scattering, and others with various capabilities [33]. Several factors currently pose a hurdle for the clinical validity of these techniques due to unstandardized protocols, variations in interpretation and poor reproducibility [33]. In general, light scattering methods, used to characterize EVs and quantify particle numbers, are not typically specific for EVs and can result in overestimating their numbers due to the co-isolation of lipoproteins and protein aggregates while other techniques may be biased with respect to specific EV size ranges, refractive index, and heterogeneity of particle populations [33].

Analysis of EV content is essential for biomarker discovery and several newer detection methods and multi-omics tools have been proposed to facilitate this research allowing for high-throughput analysis of EV cargoes to produce large-scale datasets that can be mined for biomarker signatures.

### EV protein detection and analysis

Conventional protein analysis approaches often relied on ELISA or Western Blot to evaluate EV protein biomarkers. However, these methods are characterized with low sensitivity, and low throughput as they assess a single or very few biomarkers at a time [109].

#### EV protein detection methods

To improve the sensitivity of protein detection, several techniques that involve biosensors have been recently developed and these have a wider applicability to EVs with different sizes and contents, require smaller sample volumes with less processing time, and improved readiness to be incorporated into biomedical applications [109]. Many of the new generation biosensors utilize microfluidics, immunomagnetic beads, or aptamer-based methods in their detection systems to identify and quantify EV proteins in clinical samples [25]. For instance, colorimetric and fluorescent detection in which the signal intensity is proportional to the level of the targeted EV protein have shown a clinical applicability for several biomarkers, such as PSA in human plasma using an aptamer-based sensor with a colorimetric reaction [110], a fluorescent aptasensor to detect tumor-derived exosomal proteins (EGFR, EpCAM, Her2) in plasma from breast cancer patients [111] and others [25]. A recently developed nanozyme-assisted immunosorbent assay, in which a microplate surface has been immobilized with specific capture antibodies for targeted exosomal protein markers and catalyzed a colorimetric reaction, demonstrated its clinical applicability by quantifying several exosomal proteins such as CD63, CEA, GPC-3, PD-L1 and Her2 in clinical serum samples [112]. Several recent electrochemical sensors have been proposed to enhance the sensitivity of EV protein detection by using electrochemical signals generated upon their specific binding with antibodies or aptamers to quantify the exosomes. Using this approach, electrochemical biosensors were able to detect PD-L1+ exosomes in clinical samples from breast cancer patients [113] and tumor biomarkers (EGFR, EpCAM, CD24 and GPA33) from colorectal cancer patients using a system integrated with immunocapture magnetic beads [114]. In another study, exosomes in breast cancer plasma were quantified using an integration of microfluidic device with an aptamer-based electrochemical biosensing ultrasensitive method called DeMEA [115]. Surface plasmon resonance (SPR) is another detection method based on the total reflection of light at the metal film/liquid level interface following interaction with free electrons that result in changes of light scattering intensity and spectral red shift [116]. A recent SPR platform based on transmission of SPR through periodic nanohole arrays, termed nano-plasmonic exosome sensor, was integrated with a microfluidic device for isolation of EVs [117]. In this platform, spectral changes were correlated to the level of EV target proteins enabling the identification of exosomal proteins characteristic of NSCLC such as LRG1 in urine [117]. Surface enhanced Raman scattering (SERS) has also been utilized in an affinity-based device to capture exosomes in a target-specific manner with the assistance of low-cost 3-D printed antibody array technology and demonstrated clinical applicability by detecting a significantly higher expression of exosomal Her2 and EpCAM proteins in plasma from Her2+ breast cancer patients when compared to healthy donors [118]. SERS has further shown a good performance in new potential diagnostic assays to quantify exosomal phosphoproteins in plasma of cancer patients [119] and exosomal PD-L1 in serum from NSCLC patients [120]. Aptamers that specifically target exosomal proteins have also been exploited in detection methods that use CRISPR/Cas system for PD-L1, CD109 and EGFR quantification in serum from nasopharyngeal carcinoma as potential diagnostic assays [121, 122].

#### Single EV analysis

Many of the methods used for bulk EV isolations do not account for the heterogeneity of EVs at the individual level and may contribute to low resolution and inaccurate quantification of single EVs which impede their potential adaptation [33]. As such, several techniques to overcome these challenges have been developed such as fluorescence-activated vesicle sorting [123], and high-resolution flow cytometry [124] which can more reliably quantify cancer-related protein expression and surface biomarkers over conventional flow cytometry [125]. The development of nano-flow cytometry allows a multiparametric analysis of a single EV particle as small as 40 nm in diameter, with low signal background and high sensitivity [126, 127]. Furthermore, nano-flow cytometry has demonstrated an ability to capture EV heterogeneity at the single vesicle level characterizing associated DNA and protein cargoes across different EV subpopulations in cell lines [128, 129]. Ongoing efforts are currently investigating nano-flow cytometry for the development of diagnostic cancer biomarkers as a liquid biopsy platform [130]. Other methods for single small EV detection such as super-resolution microscopy of photoactivated localization microscopy/stochastic optical reconstruction microscopy (PALM/STORM) has demonstrated potential in investigating mechanisms of exosome-mediated cancer metastasis in breast cancer cell lines [131]. Quantitative single molecule localization microscopy is another technique that demonstrated increased EGFR and CA19–9 in EVs isolated from pancreatic cancer cells compared to normal cells [132]. Droplet-based single small EV methods have shown a potential clinical application on plasma samples including droplet digital ExoELISA which detected glypican-1+ as a diagnostic biomarker for breast cancer [133], and digital profiling of proteins on individual small EVs showing that a biomarker signature of CD63, EpCAM, and MUC1 can be diagnostic for breast cancer [134]. Single particle interferometric reflectance imaging sensor is another method that provides information on the size and multiple surface biomarkers of single vesicles [135] and has recently demonstrated that tetraspanin-based capture can bias sensitivity to cancer-related EVs surface markers for diagnosis which may have clinical implications [136]. Recently, methodologies that allow single EV profiling with droplet DNA barcode sequencing to profile surface proteins on single EVs have been developed [137, 138].

#### EV proteomic profiling

Proteomics is a key to biomarker discovery, EV signature development, and investigation in clinical studies as it can comprehensively profile the expression of thousands of proteins simultaneously, ideally allowing a high coverage of the full proteome [139]. The ability of proteomics to characterize post-translational modifications (PTMs) allows the identification of biomarkers that better reflect the biological function at the cellular level and is currently being studied in EVs [140, 141]. Several MS-based bottom-up proteomics studies on clinical samples have identified EV candidate biomarkers in different cancers and these often applied protocols that were label-free [142]. Of note, a recent study which performed a comprehensive proteomic analysis of 120 plasma-derived EV and particle samples from 16 different tumor types identified cancer-specific EV protein profiles that differentiated cancer vs. normal plasma and identified a 29 EV-based protein signature related to immune function that distinguished between the 4 types of melanoma, pancreatic, lung, and colorectal cancers [143].

Multiplexed proteomic approaches which use labelling methods such as isobaric tags during sample preparation are emerging for EV analysis. Isobaric tags for relative and absolute quantification (iTRAQ) and tandem mass tags (TMT) labelling are advantageous over label-free approaches as they allow global quantification of proteins across multiple specimens in a single experiment with high throughput and reproducibility [144]. While these methods have been used to identify EV biomarkers in cell lines from multiple tumor types [22], their application to liquid biopsy clinical specimens from cancer patients was not investigated until very recently [22, 145].

In general, limitations of MS-based techniques are the use of data dependent acquisition (DDA) mode (also termed shotgun proteomics) as discovery-based approaches to perform a global proteome profiling. In DDA, the mass spectrometer automatically selects the top most abundant peptide peaks from MS1 scan to be fragmented, identified and quantified in MS2 [144] and thus could risk the reproducibility of biomarker discovery studies. Data independent acquisition (DIA) mode where the entire MS1 spectrum is collected and fragmented in MS2 overcomes this limitation and can be more reliably used for biomarker discovery and validation [146]. Targeted proteomics approaches are other emerging techniques that can be used for EV protein biomarker validation in which MS data acquisition is directed towards selecting and fragmenting specific peptides and monitoring their ions including low-abundant protein targets [144, 147]. Currently, the main targeted proteomics modes are multiple reaction monitoring (MRM) and parallel reaction monitoring (PRM) [144]. While MRM analyzes multiple fragments per peptide and can validate a limited set of predefined peptides and proteins of interest, PRM monitors all fragments in MS2 with a higher resolution. DIA and PRM have been recently used in a pioneering study to validate EV biomarkers discovered using TMT-based DDA proteomics and phosphoproteomics on plasma samples from colorectal cancer patients [148]. DDA identified 4 EV candidate proteins of fibronectin-1, haptoglobin, S100A9 and fibrinogen-α-chain (FGA) which differentiated colorectal cancer patients from healthy controls, while DIA and PRM validated FGA as the most dominant EV protein which could serve as a clinically relevant biomarker [48]. Protocols for the analysis of EV PTM proteomes including phosphoproteomics and glycoproteomics from patient plasma have also been recently described [149]. Innovative approaches combining reverse phase protein array with SEC-based EV isolation are being developed and have revealed several EV diagnostic biomarkers in plasma of breast [150] and serum of prostate [151] cancer patients with potential prognostic and predictive value. Other newer technologies such as proximity extension assay (PEA) uses oligonucleotide antibody pairs to enable high-throughput multiplexed protein detection and quantification [152]. Its commercialized platform by Olink covering 3072 PEA targets has a potential to identify biomarker panels and serve as a targeted proteomics approach for biomarker validation [152].

### EV transcriptomic analysis

RNA comprises the major nucleic acid cargo of EVs and its most prevalent species of mRNA, miRNA, and lncRNA have been intensively studied for biomarker discovery in several cancers [25, 153]. EV RNA analysis of liquid biopsy samples has been mainly investigated using qPCR, microarray, NGS [25] with a NanoString miRNA panel has recently been developed [154]. Special consideration must be taken when profiling and analyzing EV RNAs due to the abundance of small RNAs and the fact that long RNA species, such as mRNA and lncRNA, are largely present as fragments [155, 156]. NGS is the method of choice for transcriptional profiling of EVs due to its high throughput and high sensitivity allowing the detection of rare RNA species including splice variants as well as less abundant non-coding RNAs (e.g., small nuclear RNAs, circular RNAs, and RNA fragments) that can serve as potential EV RNA biomarkers [155]. Among EV RNA species, miRNAs are the most frequently investigated and represent a valuable biomarker resource due to their high stability and distinct function in mediating cellular interactions in the TME [25]. These are short single-stranded non-coding RNA molecules of nearly 19–25 nucleotides in length that inhibit the function of mRNA targets and a plethora of EV miRNA biomarkers has been identified with clinical implications in different tumor types [25, 157]. However, the annotation of miRNAs as ‘non-coding’ while other multiple annotations for the same region may be ‘protein-coding’ is a major challenge in bioinformatic analysis [145]. Currently, EV mRNAs are gaining a great interest in the field of liquid biopsy as they allow the identification of direct protein-coding targets for clinical application [158]. For instance, a study on plasma-derived EVs in gastric cancer has shown that mRNA expression levels of *CD44, PTEN,* and *FASN* using qPCR can serve as diagnostic and predictive biomarkers for treatment response [159]. Furthermore, an EV mRNA signature developed from RNA-seq serum profiling has been described to be diagnostic for prostate cancer [160].

### EV genomic analysis

When compared to cfDNA, EV DNAs are characterized by a higher stability in biofluids, are less likely to undergo non-specific degradation due to their EV lipid layer and are secreted from living rather than dying cells [161, 162]. Thus, EVs provide a particularly useful source for the discovery of highly sensitive biomarkers that can be used for monitoring disease progression such as DNA mutations in liquid biopsy. For instance, EV-based genotyping has demonstrated superiority in detecting *EGFR* mutations in different biofluids including plasma, broncho-alveolar lavage, and pleural effusion when compared to cfDNA and tissue genotyping in lung cancer [100, 163, 164]. With the advent of NGS methods and their potential application for clinical use, several whole genome sequencing studies have further demonstrated that EV-DNA can represent the whole genome of their parental cells and reflect their genetic makeup including copy number, gene fusions and mutational profiles in liquid biopsy, and thus can be used for the development of genome-derived cancer biomarkers [165]. Furthermore, targeted NGS studies on EV-DNA from liquid biopsy of various cancer types have demonstrated a high concordance in detecting specific actionable tumor mutations and tumor mutational burden to tissue-based genotyping, suggesting their utility as potential surrogate biomarkers for clinical use [161]. Similarities between EV and tissue-derived DNAs have been further reported in their methylation profiles, proposing epigenetic biomarkers with diagnostic and therapeutic implications in different tumors [161].

### EV metabolomic analysis

Metabolomics is an emerging ‘omics’ field that enables the identification and quantitation of diverse small molecules that are indicative of the metabolic state of the patient. While EV metabolites, including lipids, have been less studied in contrast to nucleic acids and proteins, growing evidence is pointing towards their role as a source for biomarker discovery, especially since lipids (e.g., cholesterol, PS, phosphatidylcholine, and phosphatidylinositol), constitute a dominant component of EVs [166]. In addition, EVs are metabolically active machines which contribute to altering the metabolism of recipient cells and thus can serve as a rich source to build metabolic profiles that differentiate between tumor and normal states as well as between different tumor stages [167]. In this regard, several EV metabolites such as steroid hormone dehydroepiandrosterone sulphate [168], ornithine amino acid, spermidine polyamine and adenosine [169], acylcarnitines [168], and a combination of three lipid species of PS 18:1/18:1, PS 16:0/18:1 and lactosylceramide (d18:1/16:0) [170], have been proposed as candidate diagnostic biomarkers in urine of prostate cancer patients. Other metabolite and lipid-based biomarkers identified using chromatography coupled with MS-based techniques on blood samples have shown a diagnostic capacity in several cancers including early-stage NSCLC [171], pancreatic [172], endometrial [173], head and neck [174], melanoma [175] and colon cancers [176]. These biomarkers have the potential to be assessed for prognosis and treatment response as chromatographic separation and MS-based methods are evolving to achieve a better metabolome coverage [166].

### EV multi-omics analyses and machine learning

The realization that EVs carry a diverse cargo of molecules which mediate intercellular signaling makes it imperative to integrate multi-omics data as a better approach for biomarker discovery than focusing only on one or few omics-based levels. The use of machine learning, a pivotal branch of artificial intelligence (AI), is now helping move forward multi-omics analyses and is increasingly being used instead of classical statistical modelling approaches to mine information and interpret high-throughput data [177]. While conventional statistics models test assumptions and draw inferences from a given dataset, machine learning builds models from unknown data and uses them to predict unidentified forthcoming measures employing algorithms such as deep learning which is more relevant to large-scale datasets [177]. For instance, one of the major successes of deep learning is predicting protein function using neural networks that perform different operations to find complex representations of data by compiling features from multiple resources such as sequence, structure, interaction networks, and scientific literature to build a model with improved predictive accuracy [178]. Integration of this information with other ‘omics’ type of data could provide a holistic view and collective insights into the mechanisms underlying patient’s response or resistance to therapy and thus aids in the selection of clinically relevant biomarkers for further validation. In the liquid biopsy research field, machine learning is progressively being used for multiplex profiling of EV biomarkers to build models that have superior performance metrics over conventional and existing diagnostic methods, resulting in more accurate classifications of various cancer types [106, 107, 143, 179–181]. For instance, Liu et al reported that a plasma EV mRNA-based signature consisting of *ESR1*, *PGR*, and *ERBB2* resulted in a more reliable diagnosis of breast cancer with the assistance of three machine learning algorithms including neural network, random forest, and support vector machine [180]. The combination of the three main biomarkers further demonstrated a better diagnostic performance than single or dual biomarker combinations [180]. Another plasma EV signature based on a weighted sum of eight protein biomarkers compiled using linear discriminant machine learning analysis distinguished metastatic breast cancer from non-metastatic or normal breast with a high accuracy and showed a potential to monitor treatment response [107]. Machine learning algorithms have also been used to build models from proteomic data that can determine cancer type of unknown primary origin. In one study, a 29 protein plasma EV-based signature that discriminated melanoma, pancreatic, lung, and colorectal cancers was discovered [143] and another study used a cross-species proteomic workflow to profile plasma EV proteins from patient-derived xenograft tumors to distinguish melanoma, pancreatic, colorectal, and breast cancers [181]. Recently, a multi-omics analysis approach using machine learning which integrated RNA-seq profiling of blood-derived exosomes of renal clear cell carcinoma along with tissue-based transcriptomic information from The Cancer Genome Atlas and single cell RNA-seq data from Gene Expression Omnibus databases revealed 13 candidate genes with high diagnostic accuracy for clear cell carcinoma [182]. Recent and ongoing studies are integrating machine learning tools to develop prognostic and predictive signatures such as those discriminating responders vs. non-responders to therapies [25]. It should be noted that one of the most common challenges of machine learning studies is that multi-omics features tested often largely outweigh the number of observation samples resulting in a ‘curse of dimensionality’(177). While different dimensionality reduction techniques can mitigate this issue, they could overfit the results to the dataset tested and even end up building a model not necessarily tailored to answer a specific clinical question. Thus, validation studies with adequately powered results using large-scale datasets are crucial to avoid compromising the reproducibility of biomarker development studies.

## EVs as mediators of intercellular crosstalk in the tumor microenvironment

**Fig. 2:**
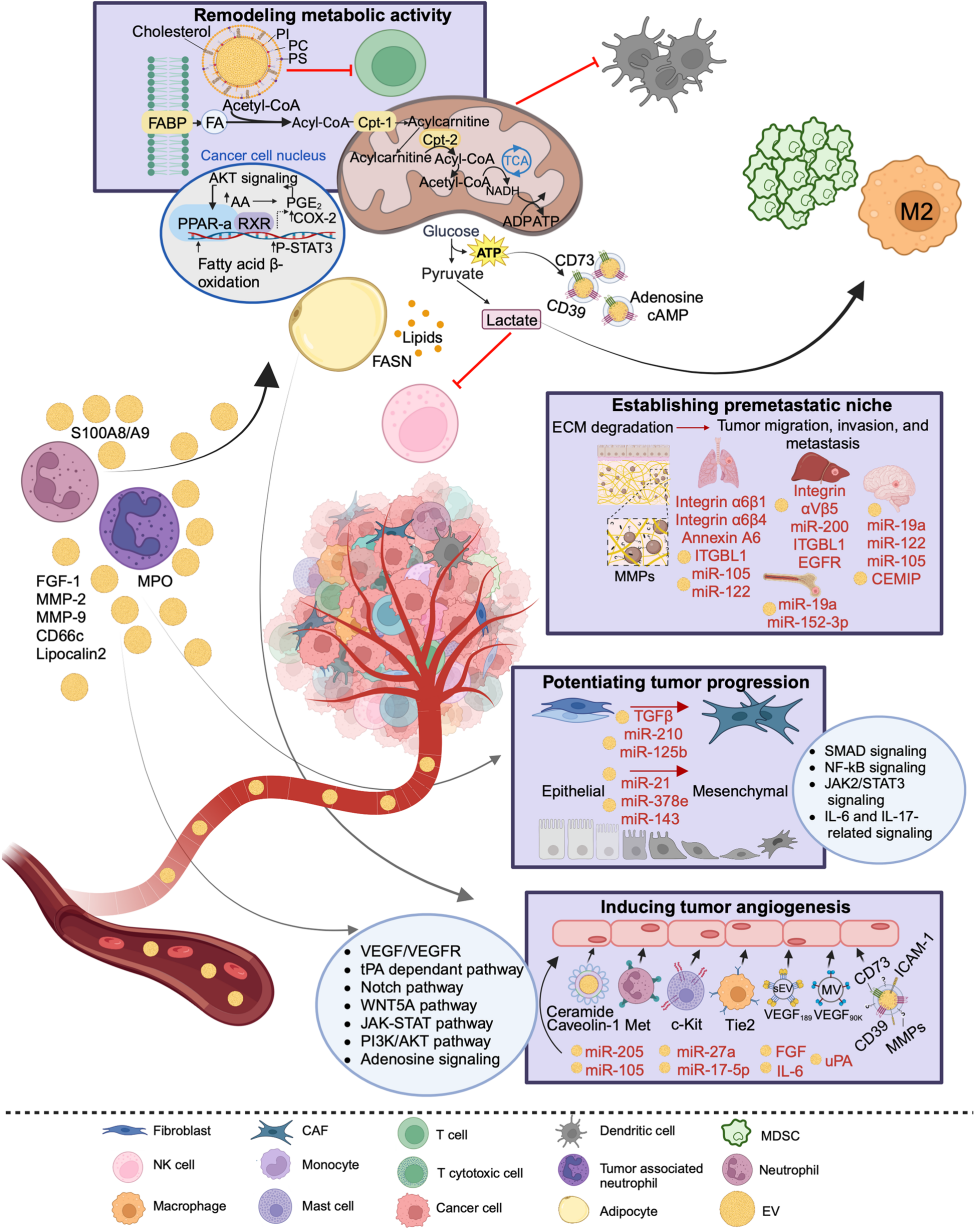
A schematic representation of the role of extracellular vesicles as mediators of intercellular crosstalk in the tumor microenvironment influencing several biological mechanisms. Abbreviations: ECM, extracellular matrix; MMP, matrix metalloproteinase; uPA, urokinase plasminogen activator; MV, microvesicle; ROS, reactive oxygen species; NO, nitric oxide; iNOS, inducible nitric oxide synthase; MDSC, myeloid-derived suppressor cell; PGE2, prostaglandin E2; HSP, heat shock protein; AA, arachidonic acid; NETs, neutrophil extracellular traps; FA, fatty acid; PS, phosphatidylserine; PC, phosphatidylcholine; PI, phosphatidylinositol; FABP, fatty acid binding protein; FGF-1, fibroblast growth factor 1; tPA, tissue plasminogen activator; PPAR, peroxisome proliferator-activated receptor. Figure was created with BioRender.com

**Fig. 3:**
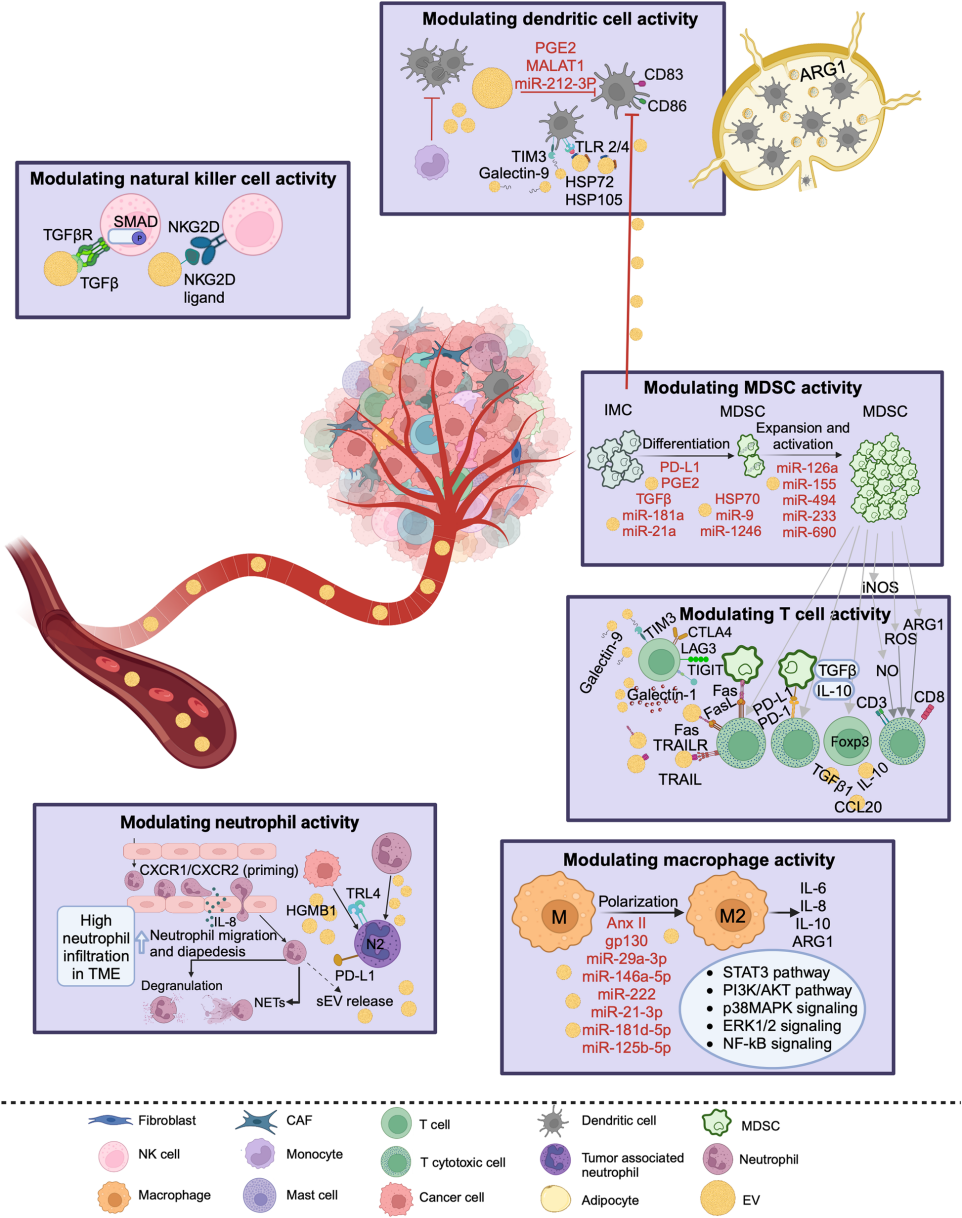
A schematic representation of the role of extracellular vesicles in regulating immune response pathways in the tumor microenvironment. Abbreviations: ROS, reactive oxygen species; NO, nitric oxide; iNOS, inducible nitric oxide synthase; MDSC**,** myeloid-derived suppressor cell; PGE2, prostaglandin E2; HSP, heat shock protein; NETs, neutrophil extracellular traps. Figure was created with BioRender.com

The recent developments in machine learning integrating multi-omics data distilled from analyzing EV cargoes underscore the importance of characterizing the biological mechanisms in the TME. Tumor-stroma interactions are dynamic and continuously alter the microenvironment to allow tumor growth [183]. EVs play a major role in mediating cellular communications in the TME, shuttling their diverse bioactive cargoes, and influencing recipient cells locally and at distant sites via activation of numerous signaling pathways [31]. Particularly, their bioactive cargoes of proteins and miRNAs have been largely described to play a role in mediating distinct biological processes in the TME [25]. While rare and less abundant non-coding RNAs, such as circulating RNAs and lncRNAs, have been investigated, miRNAs are the most heavily studied RNA species for identifying EV-based biomarkers with biological and clinical relevance. miRNAs are characterized with a high abundance, stability, and feasibility in their isolation, detection, and analysis from small volume samples [25, 184]. Interestingly, EV-associated miRNAs have been further suggested to be a more preferrable format for biomarker development than miRNAs in body fluids due to their higher stability [184]. As such, we herein summarize several EV-derived miRNAs and protein candidates that take part in mediating the intercellular crosstalk in the TME. The major biological mechanisms influenced by EVs as mediators of intercellular crosstalk in the TME are depicted in Fig. 2 and those related to regulating immune response are depicted in Fig. 3.

### EVs in potentiating tumor progression

EVs play an important role in potentiating tumor progression through the formation of tumor-promoting stroma which could involve transformation of normal fibroblasts towards tumor-promoting myofibroblasts, and cancer associated fibroblasts (CAFs). While the specific molecular mechanisms that convert normal fibroblasts to CAFs are not fully understood, EVs containing factors such as transforming growth factor beta (TGFβ) and several miRNAs have been shown to induce CAF activation and promote tumor growth [31]. For instance, EV TGFβ can trigger CAF differentiation through suppressor of mothers against decapentaplegic* (*SMAD) pathway in melanoma [185] and bladder cancer [186]. Ovarian cancer EV miR-630 has been found to activate NF-κB [187] and lung cancer EV miR-210 activated the janus kinase 2/signal transducer and activator of transcription 3 (JAK2/STAT3) pathway [188], prompting the switch of fibroblasts to CAFs. In triple negative breast cancer (TNBC), miR-125b has been reported to contribute to the development of CAFs through uptake of EVs by fibroblasts and inducing the expression of several CAF markers [189]. CAFs can originate from a variety of cells, such as epithelial cells, through the process of epithelial to mesenchymal transition (EMT). TGFβ and several miRNAs identified in CAF EVs, such as miR-21, miR-378e, and miR-143, have been reported to promote EMT and stem cell-like traits that contribute to tumor proliferation [190]. Recently, a comprehensive RNA-seq analysis on head and neck cancer cells revealed that TEVs can convert normal fibroblasts into CAF-like cells through interleukin (IL)-6 and IL-7 related signaling [191] (Fig. 2).

### EVs in establishing a premetastatic niche

EVs play a major role in remodeling the ECM to facilitate the dissemination of cancer cells from the primary tumor and to promote their colonization at distant sites [31]. EV cargo such as fibronectin has been shown to be essential for the movement of cancer cells by promoting adhesion assembly which is required for directional migration [192, 193]. In addition, EVs can induce ECM degradation by secreting proteases that degrade collagens, laminins, and fibronectin which prepare the site for a premetastatic niche [194]. The ability of EVs to further influence recipient cells by upregulating fibronectin, inducing TGFβ secretion, and activating macrophages, can contribute to the creation of a TME that promotes metastasis outgrowth [31, 195]. Interestingly, several EV subtypes have been linked to the formation of organ-specific metastatic sites, such as EVs expressing α6β4 and α6β1 integrins associated with lung metastasis, and EVs bearing αVβ5 integrin associated with liver metastasis [196]. Other EV-associated molecules, including miRNAs, surface proteins, cytokines and growth factors, have been reported to take part in altering recipient cells at future metastatic sites by modifying vascular permeability, inducing angiogenesis, facilitating immune evasion, and activating fibroblasts [31]. For instance, EVs generated by melanoma cells can induce vascular leakiness at pre-metastatic sites and reprogram bone marrow progenitors such as macrophages, neutrophils, and mast cells towards a pro-angiogenic phenotype through the receptor tyrosine kinases Tie2, Met, and c-Kit [197] (Fig. 2).

### EVs in inducing tumor angiogenesis

Angiogenesis is usually induced by the secretion of proangiogenic factors from hypoxic cancer cells which can release EVs that carry a variety of molecules that interact with endothelial cells to regulate angiogenesis [30]. Surface-bound ligands on EVs, including CD39, CD73, vascular endothelial growth factor (VEGF), MMPs, and other molecules such as fibroblast growth factors (FGF), urokinase-type plasminogen activator, and IL-6, have been particularly reported to deliver signals to receptors on endothelial cells and induce a proangiogenic response [32, 198]. Of note, VEGF isoform (VEGF_189_) bound on the EV surface has been shown to be preferentially enriched in small EVs while VEGF_90K_ is selectively enriched in microvesicles [199]. The accumulation of VEGF_189_ in small EVs has been suggested as a biomarker for disease progression and resistance to bevacizumab [200]. EVs can be internalized by endothelial cells, mainly through endocytosis, and deliver their cargo components, such as mRNA, miRNAs and angiogenic proteins, to activate relevant signaling pathways that elicit proangiogenic responses [2, 191]. These often involve activation of the VEGF/VEGFR pathway or others such as tissue plasminogen activator dependent, Notch, WNT5A, and JAK-STAT pathways [201]. EV-derived miRNAs have been reported to promote angiogenesis in several cancers, such as miR-17-5p in nasopharyngeal carcinoma via AKT/VEGF-A signaling [202], miR-205 in ovarian cancer through the PTEN/PI3K/AKT pathway [203], miR-27a in pancreatic cancer through targeting of the BTG2 tumor suppressor [204], and miR-105 in breast cancer via targeting of the tight junction protein zonula occludens-1 leading to vascular permeability [205]. EVs can further modulate endothelial cells indirectly by reprogramming other cells, via adenosine signaling, to release proangiogenic factors such as VEGF, IL-8, and angiopoietin-1 by mast cells, and thrombospondin-1 by macrophages [198]. Other bone marrow mesenchymal differentiated cells such as adipocytes can internalize TEVs enhancing their capacity to induce angiogenesis and recruit macrophages [206]. Notably, endothelial cells can transfer EVs containing caveloin-1 and ceramides to adipocytes, which in turn release EVs containing proteins and lipids that modulate signaling pathways in recipient endothelial cells, illustrating the complexity of the TME [207] Fig. 2.

### EVs in regulating tumor immune response

In general, the identification of clinically detectable cancers occurs after tumors have successfully escaped the immune system and developed immunosuppressive mechanisms that alter the surrounding microenvironment [208, 209]. These include modulation of the activity of different effector immune subsets (e.g., thymus-derived lymphocytes [T cells] and natural killer [NK] cells), and immunosuppressive cells (e.g., tumor associated macrophages, myeloid-derived suppressor cells) via cytokine and chemokine production [210]. EVs released by tumor cells play a critical role in mediating these processes and their cargoes alter to contain more immunosuppressive biomolecules as the tumor evolves to support cancer progression, angiogenesis, metastasis, and therapeutic resistance [211]. A schematic representation of the role of EVs in regulating immune response pathways in the TME is displayed in Fig. 3.

#### Modulating macrophage activity

TEVs have been shown to promote macrophage polarization towards the pro-tumoral M2 phenotype through the activation of several signaling pathways, such as PI3K/AKT, STAT3, p38 MAPK, extracellular signal-regulated kinase (ERK)1/2, and NF-κB [211]. Activation of these signaling pathways in macrophages in response to EVs can result in increased secretion of immunosuppressive molecules, such as IL-6, IL-8, IL-10, and arginase-1, and thereby enhance immune evasion, tumor progression and therapeutic resistance across different tumors [211]. M2 polarization has been reported to be further induced under hypoxic conditions in ovarian cancer in response to several EV miRNAs, such as miR-21-3p, miR-181d-5p, and miR-125b-5p, which have been proposed as potential biomarkers for therapeutic response [212]. Other EV miRNAs, including miR-29a-3p, miR-146a-5p, miR-222 [213–215] and proteins such as gp130 and AnxII [216, 217], have been linked to STAT3 activation in macrophages, leading to their M2 polarization (Fig. 3).

#### Modulating myeloid-derived suppressor cells activity

Myeloid-derived suppressor cells (MDSCs) are known to inhibit T cell activity through several biological mechanisms-including the secretion of immune suppressive enzymes and cytokines such as indoleamine 2,3-dioxygenase, arginase, IL-10, TGFβ and reactive oxygen species [218]; stimulation of apoptosis of effector immune cells via the Fas ligand pathway [218]; upregulation of PD-L1 expression, and induction of regulatory T-cell (Treg) expansion [219]. In addition, MDSCs can promote tumor angiogenesis, invasion, and metastasis, further contributing to therapeutic resistance [220]. Several components of EV cargoes and their surface markers have been demonstrated to induce the activity of MDSCs across different tumors and to stimulate MDSCs differentiation [211]. EV-associated PD-L1, prostaglandin 2 (PGE2), TGFβ, and HSP70 have been reported to be involved in the differentiation of immature myeloid cells into MDSCs [211]. This differentiation can be further stimulated via the delivery of EV miRNAs involved in activating STAT3 pathway and inhibiting its regulators such as suppressor of cytokine signaling 3 (SOCS3) and protein inhibitor of activated STAT (PIAS)3 (e.g., miR-181a and miR-9 [221]), downregulating programmed cell death protein 4 (PDCD4) protein (miR-21a) [222] and inducing dual specificity phosphatase 3 (DUSP3) enzyme activity in ERK-dependent manner (miR-1246) [223]. The function of MDSCs can be enhanced under hypoxic conditions promoting tumor growth via transfer of EV miR-29a and miR-92a [224]. MDSC-derived exosomal miRNAs, including miR-9, miR-494, miR-233, and miR-690 have been shown to increase MDSC proliferation and function [225] with miR-126a proposed as a biomarker for resistance to chemotherapy [226] (Fig. 3).

#### Modulating T cell activity

The active release of EVs by cancer cells is crucial to maintain an immune evasive TME during tumor progression and can impact T cells through a variety of mechanisms-including direct inhibition of CD8+ T cell function; stimulation of apoptosis of CD8+ T cells via the Fas ligand pathway; downregulation of signaling components related to T cell activation such as JAK3 and CD3ζ; inducing T cell exhaustion through inhibitory molecules such as PD-L1, cytotoxic T-lymphocyte-associated protein 4 (CTLA4), T cell immunoglobulin and mucin domain-containing protein 3 (TIM3), lymphocyte activation gene-3 (LAG-3), and T cell immunoreceptor with Ig and ITIM domains (TIGIT); and promoting Treg expansion [211]. Vesicular cargoes that are involved in these pathways have been reported across multiple tumors and act to hinder T cell activity, providing a rationale for their potential use as biomarkers for immunotherapeutic resistance [211]. T cell suppression and induction of Treg expansion have been shown to be facilitated through EV-associated TGFβ1, IL-10, and chemokine ligand 20 (CCL20) in breast, ovarian, prostate, colorectal and nasopharyngeal cancers [227–230]. Furthermore, the delivery of EV miRNAs, such as miR-24–3p in nasopharyngeal cancer, can prevent the activation of T helper (Th)1 and Th17 lymphocytes and convert them to immunosuppressive Treg phenotype [231]. T cell suppression can be also induced by arginase 1-containing EVs, which have been found in the ascites of ovarian cancer patients, and postulated to reduce T cell activity through uptake by DCs in lymph nodes and inhibition of antigen-specific T cell proliferation [232]. Other EV-associated molecules such as FASligand and TNF-related apoptosis-inducing ligand (TRAIL) could induce T cell apoptosis and have been found in blood samples of cancer patients including colorectal cancer and oral squamous cell carcinoma [233, 234]. Vesicular galectins such as galectin-1, which induces a suppressor phenotype in CD8+ T cells, and galectin-9, which promotes T cell apoptosis via TIM3, have been reported in colon and head and neck cancers [235] (Fig. 3).

#### Modulating dendritic cell activity

While DCs can respond to TEVs carrying tumor antigens and damage-associated molecular patterns by maturation and migration to lymph nodes and cross-presentation of antigens to major histocompatibility complex (MHC) class I to activate CD8 T cells in earlier disease stages, their function can be disrupted by EVs as the cancer progresses [211]. The ability of DCs to present antigens has been shown to be dampened in several ways by TEVs-including interaction of their TIM3 receptor with galectin-9 expressed on TEVs to promote T cell apoptosis [236]; interaction of their toll-like receptors (TLRs) 2 and 4 with HSP72 and HSP105 proteins expressed on the surface of TEVs to induce IL-6 secretion and STAT3 signaling [237]; uptake of ARG1-containing EVs by DCs in lymph nodes [232]; and their direct inhibition by various vesicular cargoes such as PGE2,metastasis associated lung adenocarcinoma transcript (MALAT) 1 protein and several miRNAs (e.g. miR-212-3p) [238–240]. Immunologically dysfunctional DCs can be further generated by an increased fatty acid oxidation induced by TEVs in the TME, which exert their effect via activating the peroxisome proliferator-activated receptor (PPAR)α, resulting in a metabolic shift towards oxidative phosphorylation of mitochondria; thus PPARα may act as a potential immunotherapeutic target [241]. TEVs can influence DC maturation via inhibition of differentiation of immature myeloid cells into DCs and shifting them towards MDSCs and M2 macrophage lineages [242]. This effect has been reported to be induced by the production of IL-6, and decreased expression of the costimulatory molecules of CD83 and CD86 [243]. Of note, PD-LI+ TEVs have been reported to suppress monocytes rather than T cells in glioblastoma resulting in an impaired maturation of DCs [244]. This finding may suggest a unique mechanism of resistance to immune checkpoint inhibitor (ICI) and the potential use of EV PD-L1 as a biomarker in the clinic (Figs. 2 and 3).

#### Modulating natural killer cell activity

Similar to the stimulatory effect on DC at earlier stages, EVs can play a role in NK cell activation by inducing the expression of stimulating receptors such as natural killer group (NKG)2D and downregulating the inhibitory receptor of CD94 to recognize tumor antigens [245]. This effect can be mediated by HSP70-enriched EVs inducing the release of GZMB, activating the TLR2/NF-κB signaling pathway and increased production of IFN-γ [246, 247]. However, the effector functions of NK cells can also be impaired by EVs as the tumor evolves via several mechanisms-including shedding of NKG2D ligand-expressing TEVs that contain MHC class I-related chain A (MICA)*008 and TGFβ1, which induce downregulation of NKG2D on NK cells [248–250]; and direct interaction of TEVs with NK cells, which reduces the expression of perforin and diminishes NK cell cytotoxic activity [251]. Of note, prolonged stimulation of NKG2D on the surface of NK cells by TEVs has resulted in inhibition of NK cells activity by promoting a higher expression of the inhibitory receptor NKG2A, and downregulation of the activating receptors NKG2D and NKp44 [245, 252]. These findings demonstrate the role of TEVs in eliciting immune cell exhaustion at later tumor stages, proposing them as a source for biomarkers related to immune resistance (Fig. 3).

#### Modulating neutrophil activity

Tumor-associated neutrophils can support pro-tumor activity and their infiltration into tumors has been associated with poor outcomes [253] and resistance to ICI [254]. EVs can carry molecular cargoes that aid in recruiting, activating, and reprogramming neutrophils towards eliciting immunosuppressive signaling in the TME [255]. For instance, the transfer of *KRAS* mutant exosomes to recipient neutrophils has been shown to upregulate IL-8 production, increase neutrophil migration, and induce the formation of neutrophil extracellular traps in colorectal cancer [256]. TEVs carrying HMGB1, which plays a role as a neutrophil recruiter, can induce a pro-tumor phenotype in neutrophils through activation of TLR4/NF-κB signaling pathway [257], activate STAT3 and upregulate PD-L1 to suppress T cells [258]. In addition, miR-146a delivered by TEVs has been linked to increased CD66+ tumor-infiltrating neutrophils, but low CD8+ T cell infiltration [259]. Neutrophils can also secrete EVs which contribute to tumor progression and creation of an immunosuppressive TME as the tumor develops [255]. While earlier stages of tumor growth involve neutrophils mediating anti-tumor effect, EVs released by neutrophils at late stages can acquire capabilities that reprogram the activity of neutrophils towards N2 phenotype which resembles M2 macrophage polarization [255]. These N2 neutrophil-derived EVs have been shown to carry several components that facilitate immune evasion, angiogenesis, tumor growth and metastasis such as FGF-1, MMP-2, MMP-9, CD66c, and lipocalin2 [255]. Of note, N2 neutrophil-derived EVs can contain high amounts of myeloperoxidase, which has been correlated to tumor progression, defective DNA repair [255], and accumulation of oxidized lipids upon the release of S100A8/9 proteins by stressed neutrophils. These can reactivate and initiate cancer cell lesions in dormant tumor cells [260], proposing a distinct mechanism for tumor recurrence and therapeutic resistance (Figs. 2 and 3).

### EVs in remodeling metabolic activity

EV metabolites are key mediators that can influence various recipient cells to help tumors meet their biosynthetic requirements and facilitate their growth in a poorly oxygenated and nutrient-deprived TME **[**30**]**. Several metabolic branches affected by EVs have been implicated in this process including amino-acids, lipids, sugars, and others such as adenosine and purines [167]. EVs play a role in remodeling fatty acid metabolism as they are enriched in bioactive lipids including cholesterol, PS, phosphatidylcholine, phosphatidylinositol [261] and others such as leukotrienes, prostaglandins, arachidonic acid and its derivative eicosanoïds [167] that have been implicated in disease progression, metastasis, and immune evasion in several tumors [262–267]. Interestingly, unsaturated diacylglycerol in EVs have been reported to be highly enriched in metastatic TNBC and to induce protein kinase D signaling pathway in endothelial cells leading to neo-angiogenesis that supports tumor progression [268]. Furthermore, inactivation of diacylglycerol by PS-expressing EVs derived from ascites of ovarian cancer patients have been shown to induce a reversible arrest in T cell receptor signaling [269]. As such, these findings present a potential EV targetable mechanism to enhance T cell immune response and overcome immunotherapy resistance. The enzyme fatty acid synthase (FASN), which is involved in de novo fatty acid biosynthesis, has been frequently identified in EVs [30, 270], suggesting that this druggable protein [271] could be a potential target. EVs have been further shown to participate in transporting various forms of fatty acids as they are enriched in transporters such as fatty acid binding protein (FABP)s, acyl-CoA binding proteins, and carnitine palmitoyltransferase 1A to mediate fatty acid oxidation [30, 169, 272]. Exosomes released by CAFs have been recently implicated in supplying tricarboxylic acid cycle metabolites, including amino acids (e.g., glutamine, arginine, glutamate, proline) and metabolites required for lipid synthesis (e.g. acetate), to promote the proliferation of cancer cells and maintain their source of energy [273]. Furthermore, EVs can contain high amounts of glycolytic enzymes which allow cancer cells to supply the metabolic requirements for their proliferation even in the presence of oxygen, known as aerobic glycolysis or Warburg effect, resulting in the production of pyruvate and lactate as final metabolites [274, 275]. The transfer of metabolic enzymes via EVs can reprogram the metabolic profiles of recipient cells, reduce the availability of glucose, and elevate free ATP and lactate levels in the TME which induce several pathways related to immune evasion and tumor growth [276]. EVs can catalyze the conversion of pyruvate to lactate since the enzyme lactate dehydrogenase has been identified in exosomes [276] and high amounts of lactic acid have been reported in EVs secreted by mesenchymal stromal cells and CAFs [273, 277]. The existence of lactic acid in the TME is known to induce the expansion of MDSCs and inhibit cytotoxic T cells and NK cells [278, 279]. In addition, free ATP in the TME can promote adenosine generation by the hydrolytic activity of EVs expressing CD39 and CD73 that induce a cAMP response in adenosine A2A receptor-positive cells resulting in T cell inhibition [280, 281]. Exosome-associated adenosine further stimulates A2B receptor positive cells to reprogram endothelial cells and macrophages towards an angiogenic phenotype and release of proangiogenic factors contributing to an immunosuppressive TME [198] (Fig. 2).

The major biological mechanisms influenced by EVs, their key biomarker candidates, and biological effect in the TME are summarized in Table 2.

**Table 2:** Summary of the major biological mechanisms influenced by extracellular vesicles, their key biomarker candidates, and biological effect in the tumor microenvironment

Biological mechanism	Key EV biomarker	Biological effect in the tumor microenvironment	References
**Potentiating tumor progression**	TGFß miR-210 miR-125b miR-630	CAF activation and promoting tumor growth.	[31, 184–190]
	miR-21 miR-378e miR-143	Promoting epithelial to mesenchymal transition and stem cell-like traits.	
**Establishing a premetastatic niche**	Fibronectin Collagens Laminins TGFβ	Extracellular matrix degradation. Tumor migration, invasion, and metastasis.	[31, 191–195]
	Integrin α6β1 Integrin α6β4 Annexin A6 ITGBL1 miR-105 miR-122	Lung metastasis	
	Integrin αVβ5 miR-200 ITGBL1 EGFR	Liver metastasis	
	miR-19a miR-122 miR-105 CEMIP	Brain metastasis	
	miR-19a miR-152-3p	Bone metastasis	
**Inducing tumor angiogenesis**	CD39 CD73 VEGF (VEGF_189_ in small EVs; VEGF_90K_ in microvesicles) MMPs FGF uPA IL-6 miR-17-5p miR-205 miR-27a miR-105	Direct interaction with endothelial cells to regulate angiogenesis. Promoting the secretion of proangiogenic factors. Reprogramming immune cells (e.g., mast cells and macrophages), via adenosine signaling, to release proangiogenic factors.	[30, 32, 197–206]
	Caveloin-1 Ceramides	Inducing the release of EVs containing proteins and lipids that modulate endothelial cells.	
**Regulating tumor immune response**			
**Modulating macrophage activity**	miR-21-3p miR-181d-5p miR-125b-5p miR-29a-3p miR-146a-5p miR-222 Anx II gp130	Promoting macrophage polarization towards the pro-tumoral M2 phenotype.	[210–216]
**Modulating myeloid-derived suppressor cells activity**	PD-L1 PGE2 TGFβ HSP70 miR-181a miR-9 miR-21a miR-1246	Inducing the differentiation of immature myeloid cells into MDSCs. ⚬ STAT3 activation and inhibition of SOCS3 and PIAS3. ⚬ Downregulation of PDCD4 protein. ⚬ Inducing DUSP3 enzyme activity in ERK-dependent manner.	[210, 220–225]
	miR-155 miR-494 miR-233 miR-690 miR-126a	Expansion and activation of MDSCs.	
**Modulating T cell activity**	TGFβ1 IL-10 CCL20 miR-24–3p Arginase 1 Fas ligand TRAIL Galectin-1 Galectin-9	Inhibition of CD8+ T-cells. Promoting Treg expansion. Inducing T cell exhaustion. ⚬ Downregulation of signaling components related to T cell activation (e.g., JAK3 and CD3ζ). ⚬ Upregulation of inhibitory molecules (e.g., PD-L1, CTLA4, TIM3, LAG-3, and TIGIT).	[210, 226–234]
**Modulating dendritic cell activity**	Galectin-9 HSP72 HSP105 ARG1 PGE2 MALAT1 miR-212-3p	Inhibition of dendritic cell antigen presentation function.	[231, 235–243]
	Fatty acids	Inducing immunologically dysfunctional dendritic cells. ⚬ Enhancing oxidative phosphorylation of mitochondria via the peroxisome proliferator-activated receptor (PPAR)α.	
	PD-L1 and other EV cargoes	Inhibition of dendritic cells maturation. ⚬ Shifting the differentiation of dendritic cells towards MDSCs and M2 macrophages. ⚬ Downregulation of costimulatory molecules of CD83 and CD86.	
**Modulating natural killer cell activity**	NKG2D ligand-expressing EVs (containing MICA*008) TGFβ1	Inhibition of NK cells activity. ⚬ Promoting a higher expression of the inhibitory receptor NKG2A. ⚬ Downregulation of the activating receptors NKG2D and NKp44.	[244–251]
**Modulating neutrophil activity**	**TEV:** *KRAS*-mutated exosomes HMGB1	Promoting neutrophil migration. Inducing the formation of neutrophil extracellular traps. Inducing a pro-tumor phenotype in neutrophils.	[252–259]
	**Neutrophil derived EVs:** FGF-1 MMP-2 MMP-9 CD66c Lipocalin2 Myeloperoxidase S100A8/9	Reprogramming the activity of neutrophils towards the pro-tumoral N2 phenotype. ⚬ Facilitating tumor progression, angiogenesis, and immune evasion.	
**Remodeling metabolic activity**	Cholesterol Phosphatidylserine Phosphatidylcholine Phosphatidylinositol Leukotrienes Prostaglandins Arachidonic acid Eicosanoïds FASN	Promoting tumor progression and metastasis. Facilitating immune evasion. ⚬ T cell inhibition.	[231, 167, 197, 260–280]
	Unsaturated diacylglycerol	Promoting neo-angiogenesis and tumor progression ⚬ Inducing protein kinase D signaling pathway in endothelial cells.	
	FASN **Fatty acid transporters:** Fatty acid binding proteins Acyl-CoA binding proteins Carnitine palmitoyltransferase 1A **CAF-associated EVs:** Tricarboxylic acid cycle metabolites, including amino acids (e.g., glutamine, arginine, glutamate, proline) and metabolites required for lipid synthesis (e.g. acetate)	Fatty acid β-oxidation. Promoting proliferation of cancer cells and maintain their source of energy. Facilitating immune evasion. ⚬ Inducing dysfunctional dendritic cells.	
	Glycolytic enzymes Lactate dehydrogenase CD39 CD73	Aerobic glycolysis / Warburg effect. Reducing the availability of glucose. Elevating free ATP and lactate levels in the TME. Inducing adenosine signaling. Facilitating immune evasion, angiogenesis, and tumor growth. ⚬ Inducing MDSC expansion. ⚬ Inhibiting cytotoxic T and NK cells. ⚬ Reprogramming macrophages and endothelial cells towards an angiogenic phenotype.	

*Abbreviations:* *EV* extracellular vesicle, *CAF* cancer associated fibroblast, *VEGF* vascular endothelial growth factor, *MMP* metalloproteinase, *FGF* fibroblast growth factor, *uPA* urokinase plasminogen activator, *PGE2* prostaglandin 2, *MDSC* myeloid-derived suppressor cell, *HSP* heat shock protein, *SOCS3* suppressor of cytokine signaling 3, *PDCD4* programmed cell death protein 4, *ERK* extracellular signal-regulated kinase, *DUSP3* dual specificity phosphatase 3, *CCL20* chemokine ligand 20, *TRAIL* TNF-related apoptosis-inducing ligand, *CTLA4* cytotoxic T-lymphocyte-associated protein 4, *TIM3* T cell immunoglobulin and mucin domain-containing protein 3, *LAG-3* lymphocyte activation gene-3, *TIGIT* T cell immunoreceptor with Ig and ITIM domains, *NK* natural killer, MICA*008 MHC class I-related chain A*008, *NKG2D* natural killer group 2D, *TEV* tumor derived extracellular vesicle, *ATP* adenosine triphosphate**,** *TME* tumor microenvironment

## Clinical application of EVs as predictive biomarkers for immunotherapy: insights from NSCLC and melanoma studies

The involvement of EVs in transferring a plethora of biomolecules that shape the TME and modify drug response could have great clinical implications. While most EV biomarkers published to date are diagnostic, demonstrating their prognostic and especially predictive capacity is essential prior to their translation into clinic. Recently, several predictive EV biomarkers have been investigated across different tumors and analyzed in the context of newer therapies, specifically immunotherapy, as the leading targeted agents being integrated in the standard-of-care paradigm. Indeed, many of the EV biomarkers tested are involved in biological mechanisms that support their association with therapeutic response or resistance to immunotherapy and shed the light on immune-evasive mechanisms that could explain the modest response rates observed in cancer patients [2, 282, 283]. Recent attempts have been made to identify EV-based biomarkers that can be linked to therapeutic response using liquid biopsy from cohorts of patients treated with ICI, particularly in NSCLC and melanoma (Table 3). Most of these studies have focused on miRNAs and proteins in EVs because of the privilege of enhanced technical miRNA detection and protein analyses [184]. Among RNA species, miRNAs are the most investigated for predictive biomarker development in the clinical setting due to their high abundance, stability, feasibility of analysis, and distinct functions in mediating cellular interactions in the TME [25]. Thus, EV-based protein and miRNA predictive biomarkers for immunotherapy response served as the focus of this review.

**Table 3:** Key clinical studies assessing extracellular vesicle blood-based predictive biomarkers for immunotherapy in NSCLC and melanoma

Cancer type	Cohort analyzed	Key EV biomarkers	Key clinical findings (Good vs. poor response)
NSCLC Shukuya et al. 2020 [284]	Plasma (*n* = 29)	miR-200c-3p, miR-21-5p, miR-28-5p	Association with poor response. AUC for the combination (miR-21-5p, miR-28-5p and miR-199a-3p) = 0.925; AUC for (PD-L1 tissue expression) = 0.575
NSCLC Peng et al. 2020 [285]	Plasma (*n* = 30)	miR-320d, miR-320c, miR-320b, miR-125b-5p	Association with poor response. Association with progressive disease compared to partial response for baseline levels. Reduction in miR-125b-5p post-treatment levels when compared to pre-treatment samples among those who achieved a partial response.
NSCLC Miguel-Perez et al. 2022 [286]	Plasma (*n* = 72) Training (*n* = 33) Validation (*n* = 39)	Δ PD-L1	Association with poor response, shorter PFS and OS for the increase in EV PD-L1 following treatment with immunotherapy.
NSCLC Brocco et al. 2021 [287]	Whole blood (*n* = 59)	CD31+ endothelial-derived EVs 10 proteins enriched for neutrophil degranulation (e.g., annexin A2 and S100A8/9)	Association with poor response Low blood concentration of CD31+ endothelial-derived EVs pre-treatment was associated with longer OS and higher disease control. EV-associated proteins involved in neutrophil degranulation (e.g., annexin A2 and S100A8/9) decreased during treatment in responders while a positive change was observed among non-responders.
Melanoma And NSCLC Del Re et al. 2018 [288]	Plasma Melanoma cohort (*n* = 18) NSCLC cohort (*n* = 8)	Δ *PD-L1* (mRNA)	Association with poor response for the increase in EV *PD-L1* following treatment with immunotherapy. Association with complete and partial responses for the decrease in EV *PD-L1* following treatment with immunotherapy.
Melanoma Chen et al. 2018 [289]	Plasma (*n* = 44)	PD-L1	Association with poor response for pre-treatment plasma EV PD-L1 protein levels. Association with improved response for the increase in EV PD-L1 among responders. This observation was not found among non-responders.
Melanoma Cordonnier et al. 2020 [290]	Plasma (*n* = 46)	Δ PD-L1	Association with poor response, PFS and OS especially in an increase of EV PD-L1 > 100 pg/mL post immunotherapy. EV PD-L1 was detected in all patients (100%) whereas only 67% were PD-L1 positive in tumor biopsies. AUC for Δ PD-L1 = 0.87 for discriminating between responders vs. non-responders.
Melanoma (Turiello et al. 2022 [291])	Serum (*n* = 41)	PD-L1 CD73	Association with improved response for the increase in EV PD-L1 among responders. Association with poor response for the increase in EV CD73 among non-responders.
Melanoma (Shi et al. 2020 [292])	Plasma Training (*n* = 50) Validation (*n* = 30)	Dynamics of several on-treatment transcripts and enriched pathways based on RNA-seq analysis (e.g., T cell receptor, CD28 costimulatory and CTLA4 signaling)	Association with decreased activity during the receipt of immunotherapy in non-responders. Association with poor response for transcripts such as (e.g., CD1A, MAP2K4, TRBV7–2, IGFL1) in pre-treatment samples of non-responders.
Melanoma Tucci et al. 2017 [293]	Plasma (*n* = 59)	EV biomarkers from T-cells (P-D1 and CD28) and dendritic cells (CD80 and CD86) based on flow-cytometry analysis	Association of baseline EV P-D1 and CD28 from T cells with improved PFS and OS. Upregulated levels of costimulatory molecules (CD80 and CD86) on dendritic cells at the end of immunotherapy treatment in patients who achieved a longer PFS.
Melanoma Serratì et al. 2022 [294]	Plasma (*n* = 71)	EV biomarkers from T-cells (P-D1) and melanoma cells (PD-L1)	Association of higher levels of P-D1+ EVs from CD8+ T cells with poor response, PFS, and OS. Association of higher levels of PD-L1+ EVs from melanoma cells with poor response, PFS, and OS. AUC = 0.86 for the combination of (P-D1+ EVs from CD8+ T cells and PD-L1+ EVs from melanoma cells) showing a strong predictive value for poor PFS.

*Abbreviations:* *EV* extracellular vesicle, *NSCLC* non-small-cell lung cancer, *PD-L1* programmed death-ligand 1, *AUC* area under the curve, *PFS* progression free survival, *OS* overall survival, *CD* cluster of differentiation

In lung cancer, elevated levels of miR-200c-3p, miR-21-5p, and miR-28-5p have been reported in pre-treatment plasma EVs from advanced NSCLC patients who did not respond to anti-PD1 or anti-PDL1 therapy [284]. Furthermore, the combination of the three biomarkers of miR-199a-3p, miR-21-5p, and miR-28-5p yielded better performance metrics in predicting response to immunotherapy than PD-L1 immunohistochemical (IHC) assessment [284]. Specific miRNAs at baseline (miR-320d, miR-320c, and miR-320b) have been further described to predict progressive disease vs. partial response to ICI in NSCLC [285]. In addition, miR-125b-5p, which acts as a T cell suppressor, was significantly reduced in post-treatment plasma EVs when compared to pre-treatment samples among those who achieved a partial response to ICI [285]. Interestingly, recent studies investigating EV protein biomarker dynamics in NSCLC have reported an increase in EV PD-L1 following treatment with ICI to be associated with poor response and unfavourable survival outcomes [286]. In support of this clinical finding, EVs expressing PD-L1 derived from lung cancer cells have been shown to reduce T cell activity and promote tumor growth, and thus were proposed as a critical mediator of immune escape [295]. Low blood concentrations of pre-treatment CD31+ endothelial-derived EVs were found to be associated with longer overall survival and higher disease control on ICI [287]. Other EV-associated proteins involved in neutrophil degranulation (e.g., annexin A2 and S100A8/9) were found to decrease during ICI treatment in responders while a positive change was observed among those who did not achieve response [287]. Consistent with these findings, a recent study linked poor response to ICI in NSCLC with on-treatment changes in plasma proteins related to neutrophil function [296].

In melanoma, dynamic changes in PD-L1 levels have been the focus of RNA and protein analysis of EV biomarkers related to ICI response [157]. A study evaluating *PD-L1* mRNA expression in plasma derived EVs to monitor therapeutic response in melanoma and NSCLC has reported decreased *PD-L1* levels among patients who achieved partial and complete response while increased expression of EV *PD-L1* was observed among non-responders following treatment with ICI [288]. Recent studies have demonstrated that pre-treatment plasma EV PD-L1 protein levels were significantly higher among metastatic melanoma patients who did not respond to ICI [289, 294]. In contrast, increased levels of EV PD-L1 during early treatment with immunotherapy was found to predict improved response rates in melanoma patients as described by Chen et al [289]*.* Interestingly, this association was not observed in non-responders and thus findings of this study suggest that EV PD-L1 may have different clinical implication depending on sampling times, the course of disease, treatment schedules and among responders vs. non-responders. In this context, the authors suggested that baseline high levels of EV PD-L1 may indicate a T cell exhaustion, while increased levels of EV PD-L1 levels following immunotherapy could be related to reinvigoration of T cells and an improved anti-tumor immune response which is more pronounced in responders as they have a ‘less exhausted’ pre-existing tumor immunity when compared to non-responders whose T cells can no longer be recovered with immunotherapy [157, 289]. Similar findings have been reported by Turiello et al showing that early on-treatment serum levels of EV PD-L1 have increased in responders to immunotherapy, while this observation was not reported in non-responder melanoma patients [291]. In contrast, the expression of CD73 on EVs, which produces adenosine and contributes to T-cell suppression, was found to increase early on-treatment among melanoma patients who failed to respond to ICI [291], suggesting the potential clinical relevance of assessing molecules beyond PD-L1 during treatment with ICI. Interestingly, another study has further shown that while EV PD-L1 pre-treatment levels were not predictive for immunotherapy response, their changes after treatment, specifically with an increase of EV PD-L1 > 100 pg/mL post ICI, were predictive of poor survival outcomes. Furthermore, while EV PD-L1 was detected in all patients in the study, only 67% were PD-L1 positive in tumor biopsies [290]. These results highlight the known imperfect predictive capacity of IHC PD-L1 as a biomarker for immunotherapy response [297] and support further investigation of plasma EV PD-L1 as an emerging predictive biomarker in the clinic.

Recent efforts have been made to identify EV biomarkers related to immunotherapy response in melanoma using NGS. Shi et al. performed a comprehensive RNA-seq profiling of plasma EVs obtained from metastatic melanoma patients reporting a decrease in several on-treatment transcripts and pathways related to T cell receptor, CD28 costimulatory and CTLA4 signaling during the receipt of ICI in non-responders [292]. Furthermore, multiple transcripts known to be associated with immunotherapy resistance (e.g., *CD1A, MAP2K4, TRBV7–2, IGFL1*) were found to be enriched in pre-treatment samples of non-responders [292]. EVs released from immune cells have been also reported as a source for biomarkers related to ICI response. Higher levels of PD1+ EVs, specifically from CD8+ T cells, were found to be strongly correlated with poor progression free survival (PFS) among metastatic melanoma patients treated with PD1 inhibitors [294]. In this study, Serratì et al further investigated the mechanism by which EVs were directly involved in resistance to anti-PD1 drugs and found that circulating PD1+ EVs can bind the anti-PD1 nivolumab as demonstrated by its conjugation with a fluorescent tag (fluo-nivolumab) [294]. These findings suggest that PD1+ EVs binding nivolumab can directly neutralize the therapeutic effect of anti-PD1 resulting in an impairment of PD1/PD-L1 inhibition by competing with cell surface bound molecules for their binding partners [294]. However, another study by Tucci et al*,* which performed an analysis by flow-cytometry in metastatic melanoma patients treated with ipilimumab, has reported baseline EV PD1 and CD28 from T cells to be associated with improved PFS and overall survival [293]. The conflicting findings between these two studies regarding the predictive capacity of EV PD1 for immunotherapy response might be explained by the differences in the mechanism of action by which nivolumab vs. ipilimumab inhibit T cell activity. Based on the findings from Serratì et al, it might be postulated that since ipilimumab targets CTLA4 rather than PD1, it does not bind to circulating PD1+ EVs as nivolumab and thus no competition with cell surface bound molecules of PD1 exists. However, further studies are needed to better characterize the predictive capacity of EV PD1 in relation to various regimens of immunotherapy.

It should be noted that analysis of additional EV-associated immune subsets, such as the levels of costimulatory molecules of CD80 and CD86 on DCs, were found to be upregulated at the end of ICI treatment in patients who achieved a longer PFS [293]. Incorporating these results in the context of recent attempts to study the dynamic change of other immune cells in blood may build a better predictive model related to immunotherapy response. Recently, Gaißler et al reported that an increase in peripheral MDSC counts under ICI, specifically with a cut-point of monocytic MDSC counts > 18.1%, predicted ICI resistance in metastatic melanoma [298]. However, pre-treatment elevated frequencies of peripheral monocytic MDSCs were not predictive, suggesting that the dynamic change under therapy is a better predictor of clinical benefit in patients with metastatic melanoma [298]. Altogether, these studies illustrate the contribution of several biological mechanisms to exert immunotherapy response and propose integrating multiple blood-based biomarkers to guide clinical management.

The role of PD-L1 as a biomarker for immunotherapy response in its EV-associated form has been shown to display different predictive associations than soluble PD-L1 in multiple tumors [299]. While several studies have linked EV PD-L1 with resistance to ICI in melanoma [289, 294, 299], this association was not clearly found with soluble PD-L1 which has been even linked to a greater likelihood of developing a partial response on CTLA4 blockade after five months of treatment [300]. The higher immunosuppressive impact of EV-associated PD-L1 compared to the soluble form has been suggested to be a result of the essential role of MHC molecules expressed on EVs which interact with T cell receptors and enhance the inhibitory effect of EV PD-L1 to T cells [299]. Indeed, gastric cancer cell line-secreted exosomal PD-L1 has been found to mechanistically induce stronger T cell dysfunction compared with its soluble counterpart due to MHC-I expression [301] and multiple studies across different tumor types have consistently illustrated that EV PD-L1 can inhibit T cell activity and promote tumor growth [299]. In addition, the correlation between EV PD-L1 and T cell counts or cytokines in plasma of gastric cancer patients has been reported to be negative pointing towards its immunosuppressive effect [301], while a positive correlation between EV PD-L1 and TGFβ1 has been described [302]. The plasma/serum levels of PD-L1 expressed on EVs, rather than soluble PD-L1, have been further associated with disease progression and clinicopathological features in NSCLC, and head and neck tumors [303, 304]. Interestingly, in a phase I clinical trial (NCT01935921) which enrolled 18 head and neck squamous cell carcinoma patients to receive the anti CTLA4 of ipilimumab in addition to standard combination of cetuximab and radiation therapy, the dynamics of both tumor and T cell derived exosomes pre, during and post therapy were found to predict recurrence [305]. Specifically, exosomes derived from the immune subsets of Treg (CD3+/CD15s+) and (CD3−/PD-L1+) were found to increase from the baseline levels among patients who experienced recurrence. In contrast, Treg (CD3+/CD15s+) exosomes stabilized and (CD3+/CTLA4+) exosomes declined after ipilimumab therapy among those who remained disease free [305]*.* These findings highlight the role of plasma EVs derived from both tumor and immune cells for a better stratification of patients’ response to immunotherapy.

Currently, immunotherapy clinical trials integrating the assessment of EV liquid-biopsy biomarkers are underway in various tumors such as EV PD-L1 and miRNAs in NSCLC (NCT04427475), PD-L1 in melanoma (NCT05744076), PD-L1 and exosomes in colon cancer (NCT03927898; NCT04483219), PD-L1 and CD20 in diffuse large B cell lymphoma (NCT03985696), PD-L1 and LAG-3 in HCC (NCT05575622), exosomes in TNBC (NCT02977468), exosomes and their RNA in renal cell carcinoma (NCT05705583) (Table 4).

**Table 4:** Selected ongoing immunotherapy clinical trials assessing extracellular vesicle liquid biopsy-based biomarkers in different cancers

Clinical Trials.Gov Identifier	Cancer type, Origin of exosomes	EV biomarker tested	Description
NCT04427475	NSCLC (Plasma)	PD-L1 miRNAs	Changes of PD-L1 and miRNAs expression on exosomes in plasma of NSCLC patients before and after treatment with immunotherapy
NCT03927898	Colon cancer (Blood)	PD-L1	Changes of PD-L1 expression on exosomes in peripheral blood after treatment with the anti-PD1 toripalimab
NCT04483219	Colon cancer (Serum)	Exosomes	Exosomes from serum samples of patients during treatment with tyrosine kinase inhibitor in combination with anti-PD1 among microsatellite stable metastatic colorectal cancer patients
NCT03985696	Diffuse large B-cell lymphomas (Plasma)	PD-L1 CD20	PD-L1 and CD20 on exosomes from plasma of diffuse large B-cell lymphomas patients treated with immunotherapy
NCT05575622	Hepatocellular carcinoma (Blood)	PD-L1 LAG-3	PD-L1 and LAG-3 on exosomes from blood of hepatocellular carcinoma patients treated with immunotherapy
NCT02977468	Triple negative breast cancer (Serum)	Exosomes	Serum exosomes in treatment-naive triple negative breast cancer patients receiving pembrolizumab in addition to intraoperative radiation therapy
NCT05744076 (EXOMEL1)	Melanoma (Plasma)	PD-L1	PD-L1 on exosomes from plasma of melanoma patients before and after treatment with immunotherapy Comparing PD-L1 labeling in exosomes to PD-L1 labeling in plasma and in tumor tissue
NCT05705583	Renal cell carcinoma (Blood and urine)	Exosomes Exosomal RNA	Exosomes concentration and their RNA from blood and urine of renal cell carcinoma patients treated with immunotherapy

*Abbreviations:* *EV* extracellular vesicle, *NSCLC* non-small-cell lung cancer, *PD-L1* programmed death-ligand 1, *LAG-3* lymphocyte activation gene-3, *CD20* cluster of differentiation 20

## Conclusions and future perspectives for integrating EV biomarkers into clinical trials

While the field of precision oncology is rapidly expanding and more immunotherapy options are revolutionizing cancer treatment paradigms, therapeutic resistance remains a pressing challenge [2]. The recognition that EVs play a critical role in mediating immune evasion while still being easily obtained from a routine, minimally-invasive, and convenient liquid biopsy-based test, has recently led to the emergence of several clinical trials using them as a source for biomarker development. Indeed, many of the cellular communications and pathway crosstalk in the TME, mediated by the diverse bioactive cargoes of EVs we summarized in this review, can explain the high rates of failures still seen with immunotherapy and subsequently can be exploited for tailoring better therapeutic strategies. In this context, improving response rates to immunotherapy would rely on generating an effective anti-tumor immune response by targeting EV-associated activities in the complex immunosuppressive milieu of TME spanning from tumor cells, CAFs, vascular endothelial cells, and several immunosuppressive cells such as MDSCs, Treg cells and others. These components are not typically revealed by IHC PD-L1 clinical tests and may imply a different predictive role for EV-associated PD-L1 and other EV biomolecules that govern the interaction between T cells and intercellular pathways to exert their immunosuppressive effect. For example, a recent innovative study using a system called extracellular vesicle-target cell interaction detection through SorTagging (ETIDS) has shown that the interaction of tumor-derived EV PD-L1 with PD1 on T cells relies on the expression of the intercellular adhesion molecule 1 (ICAM-1) on EVs [306]. Blocking this molecule significantly reduces such interaction, suggesting that ICAM-1 is a pre-requisite for EV PD-L1 mediated suppressive effect [306]. Other EV molecules may also play an essential role mediating the interaction of TEVs with T cells and these can serve as potential therapeutic targets. Deciphering biological mechanisms mediated by EVs can further spur recognition of strategies to block resistance to several therapeutic options beyond immunotherapy. For instance, EVs can package increasing quantities of VEGF in tumors enriched for angiogenesis, making them not accessible to anti-VEGF antibodies, and triggering intracrine VEGF signaling in endothelial cells that mediate resistance to anti-VEGF therapy [306]. Altogether, these studies underscore the importance of identifying EV-based biomarkers for the design of approaches that overcome resistance to several drugs. In addition, they highlight the impact of leveraging the ability to modify EV cargoes to develop EV-based treatments and carriers targeting distinct biological mechanisms as a newer way of personalized medicine [31, 157, 307].

Though the clinical application of candidate EV molecules as predictive biomarkers to monitor immunotherapy response has been mainly demonstrated in NSCLC and melanoma, this is still a work in progress and more validation studies are needed prior to their translation into clinic. Further pre-clinical studies that characterize targetable pathways influenced by EVs could also provide a proof-of-concept that informs better clinical trial design and therapeutic strategies tailored to specific tumor types or even subtypes. Integrating such findings with multi-omics analyses through the support of pioneering machine learning algorithms can distill the most relevant and often dominant candidate biomarkers and pathways, providing reliable insights into the mechanisms underlying patient’s response or resistance to therapy. Such information can further account for the heterogeneity that exists across different tumor types and when correlated with matched tissue can aid building AI-assisted EV-based profiles for different tumors that directly link to clinical outcomes. Incorporating these profiles in the context of current emerging technologies, such as single cell analysis and spatial profiling, holds great promise into building a holistic view of the TME where some tumor tissue interactions can be mirrored in liquid biopsy, while others expand opportunities for exploring newer strategies. In this regard, recent advances in single EV analysis that can be applied to a variety of EV sources and isolation techniques can aid characterizing the individual-level heterogeneity of EVs, identify subsets for clinical application, and complement ecosystem-based patient classifications on tissue.

Overall, the advent of high-throughput omics techniques where thousands of candidate EV biomolecules are simultaneously quantified and evaluated, coupled with recent improvements to EV isolation and detection, can more reliably facilitate the discovery of biomarkers, classifiers, and signatures that can be translated as clinical tests. However, several analytical factors still impede this process, mainly due to lack of standardized EV isolation protocols, variability in interpretation methods, and poor reproducibility often resulting from a smaller sample size and limited statistical power which require additional cohorts for validation. Due to these issues and as EV studies are increasingly conducted to discover and validate biomarkers for clinical use, developing criteria that ensure accurate, complete, and transparent reporting for both analytical and clinical validity of EV biomarkers is highly warranted. Recent initiatives to create EV databases that integrate several levels of biology, detailed methodologies, analyses techniques and characteristics of cohorts tested are blazing the trail to the development of such criteria. EV-track (http://evtrack.org) [308] is a knowledgebase currently endorsed by ISEV to enhance rigor and reproducibility in EV studies, consistent with the MISEV guidelines [33].

In conclusion, improving survival rates and overcoming therapeutic resistance to immunotherapy is an unmet clinical need and prevails as a priority for the immuno-oncology research community [2]. EV-based biomarkers may aid resolving many questions that are not answered by tissue-based markers to decipher immune resistance mechanisms. Indeed, the EV research field is setting the ground to the development of biomarkers likely to be translated into clinical assays and incorporated into clinical trials. Although still in their early stages, the emerging advances being seen with EV research studies are promising to transform the burgeoning field of precision immuno-oncology soon and improve patients’ outcomes.

## Data Availability

Not applicable.

## Abbreviations

ARG1: arginase 1
Akt: Ak strain transforming
AI: artificial intelligence
ATP: adenosine triphosphate
CAF: cancer associated fibroblast
CCL20: chemokine ligand 20
CD: cluster of differentiation
CEA: carcinoembryonic antigen
cfDNA: circulating cell-free DNA
CTC: circulating tumor cell
ctDNA: circulating tumor DNA
CRISPR: clustered regularly interspaced short palindromic repeats
CTLA4: cytotoxic T-lymphocyte-associated protein 4
DC: dendritic cell
DDA: data dependent acquisition
DIA: data independent acquisition
DUSP3: dual specificity phosphatase 3
ECM: extracellular matrix
EGFR: epidermal growth factor receptor
ELISA: enzyme-linked immunosorbent assay
EMT: epithelial to mesenchymal transition
ERK: extracellular signal-regulated kinase
ESCRT: endosomal sorting complex required for transport
EpCAM: epithelial cellular adhesion molecule
ETIDS: extracellular vesicle-target cell interaction detection through SorTagging
EVOD: Extracellular vesicles on demand
EV: extracellular vesicle
ExoCAS-2: exosome clustering and scattering-2
FABP: fatty acid binding protein
FASN: fatty acid synthase
FGA: fibrinogen-α-chain
FGF: fibroblast growth factor
GPA33: glycoprotein antigen 33
GPC-3: glypican-3
GZMB: granzyme B
HCC: hepatocellular carcinoma
Her2: human epidermal growth factor receptor 2
HSP: heat shock protein
ICAM-1: intercellular adhesion molecule 1
ICI: immune checkpoint inhibitor
IFN-γ: interferon-γ
IHC: immunohistochemical
IL: interleukin
ILV: intraluminal vesicle
ISEV: International Society of Extracellular Vesicles
iTRAQ: isobaric tags for relative and absolute quantification
JAK: janus kinase
*KRAS*: Kirsten rat sarcoma virus
LAG-3: lymphocyte activation gene-3
lncRNA: long non-coding RNA
MALAT: metastasis associated lung adenocarcinoma transcript
MDSC: myeloid-derived suppressor cell
MHC: major histocompatibility complex
microRNA: miRNA
MISEV: minimal information for studies of extracellular vesicles
MMP: metalloproteinase
MRM: multiple reaction monitoring
MS: mass spectrometry
MV: microvesicle
MVB: multivesicular body
NGS: next generation sequencing
NK: natural killer
NKG: natural killer group
NTA: nanoparticle tracking analysis
NSCLC: non-small-cell lung cancer
PALM: photoactivated localization microscopy
PD1: programmed death 1
PDCD4: programmed cell death protein 4
PD-L1: programmed death-ligand 1
PEA: proximity extension assay
PEG: polyethylene glycol
PFS: progression free survival
PGE2: prostaglandin 2
PIAS3: protein inhibitor of activated STAT 3
PI3K: phosphoinositide 3-kinase
PPAR: peroxisome proliferator-activated receptor
PRM: parallel reaction monitoring
PS: phosphatidylserine
PTMs: post-translational modifications
qPCR: quantitative reverse transcription polymerase chain reaction
SEC: size exclusion chromatography
SERS: surface enhanced raman scattering
SMAD: suppressor of mothers against decapentaplegic
SOCS3: suppressor of cytokine signaling 3
SPR: surface plasmon resonance
STAT: signal transducer and activator of transcription
STORM: stochastic optical reconstruction microscopy
TEV: tumor derived extracellular vesicle
TGFβ: transforming growth factor beta
TIGIT: T cell immunoreceptor with Ig and ITIM domains
TIM3: T cell immunoglobulin and mucin domain-containing protein 3
TLR: toll-like receptor
TME: tumor microenvironment
TMT: tandem mass tags
TNBC: triple negative breast cancer
TRAIL: TNF-related apoptosis-inducing ligand
Treg: T regulatory
uPA: urokinase plasminogen activator
VEGF: vascular endothelial growth factor
